# Effects of Intermittent Alcohol Exposure on Emotion and Cognition: A Potential Role for the Endogenous Cannabinoid System and Neuroinflammation

**DOI:** 10.3389/fnbeh.2017.00015

**Published:** 2017-02-07

**Authors:** Laura Sanchez-Marin, Francisco J. Pavon, Juan Decara, Juan Suarez, Ana Gavito, Estela Castilla-Ortega, Fernando Rodriguez de Fonseca, Antonia Serrano

**Affiliations:** Unidad de Gestion Clinica de Salud Mental, Instituto de Investigacion Biomedica de Malaga, Hospital Regional Universitario de Malaga, Universidad de MalagaMalaga, Spain

**Keywords:** rats, intermittent alcohol, adolescence, endocannabinoid system, neuroinflammation, anxiety, brain, recognition memory

## Abstract

Intermittent alcohol exposure is a common pattern of adolescent alcohol use that can lead to binge drinking episodes. Alcohol use is known to modulate the endocannabinoid system (ECS), which is involved in neuronal communication, neuroplasticity, neuroinflammation and behavior. Adolescent male Wistar rats were exposed to 4-week intermittent alcohol intoxication (3 g/kg injections for 4 days/week) or saline (*N* = 12 per group). After alcohol deprivation, adult rats were assessed for emotionality and cognition and the gene expression of the ECS and other factors related to behavior and neuroinflammation was examined in the brain. Alcohol-exposed rats exhibited anxiogenic-like responses and impaired recognition memory but no motor alterations. There were brain region-dependent changes in the mRNA levels of the ECS and molecular signals compared with control rats. Thus, overall, alcohol-exposed rats expressed higher mRNA levels of endocannabinoid synthetic enzymes (N-acyl-phosphatidylethanolamine phospholipase D and diacylglycerol lipases) in the medial-prefrontal cortex (mPFC) but lower mRNA levels in the amygdala. Furthermore, we observed lower mRNA levels of receptors CB_1_ CB_2_ and peroxisome proliferator-activated receptor-α in the striatum. Regarding neuropeptide signaling, alcohol-exposed rats displayed lower mRNA levels of the neuropeptide Y signaling, particularly NPY receptor-2, in the amygdala and hippocampus and higher mRNA levels of corticotropin-releasing factor in the hippocampus. Additionally, we observed changes of several neuroinflammation-related factors. Whereas, the mRNA levels of toll-like receptor-4, tumor necrosis factor-α, cyclooxygenase-2 and glial fibrillary acidic protein were significantly increased in the mPFC, the mRNA levels of cyclooxygenase-2 and glial fibrillary acidic protein were decreased in the striatum and hippocampus. However, nuclear factor-κβ mRNA levels were lower in the mPFC and striatum and allograft inflammatory factor-1 levels were differentially expressed in the amygdala and hippocampus. In conclusion, rats exposed to adolescent intermittent alcohol displayed anxiety-like behavior and cognitive deficits in adulthood and these alterations were accompanied by brain region-dependent changes in the gene expression of the ECS and other signals associated with neuroinflammation and behavior. An intermittent adolescent alcohol exposure has behavioral and molecular consequences in the adult brain, which might be linked to higher vulnerability to addictive behaviors and psychopathologies.

## Introduction

Alcohol is the most widely used recreational drug, and its consumption is increasing in young people and adolescents. A common pattern of alcohol intake among adolescents is the intermittent alcohol exposure, which can lead eventually to heavy episodic drinking. Thus, adolescent binge drinking is a major public health concern and it is associated with long-term health consequences, including mental problems (e.g., anxiety, mood and personality disorders) and substance use disorders, primarily alcoholism (Oesterle et al., 2004; Dawson et al., 2008; Read et al., 2008). Further, the potential role of alcohol use as a risk factor for adult psychiatric disorders cannot be discarded.

The molecular actions of alcohol on the brain are complex, and involve several mechanisms and signaling systems and some of these actions occur in both the adult and the adolescent brain. Brain maturation mainly happens during adolescence, when numerous plastic and dynamic processes are happening, and the vulnerability of the developing brain to the toxic effects of alcohol is higher. Therefore, early alcohol exposure can produce alterations in the brain structure and function, resulting in behavioral and cognitive deficits (Nagel et al., 2005; Zeigler et al., 2005; Guerri et al., 2009). These long-term harmful effects of alcohol exposure on behavior have also been confirmed in animal models (e.g., learning dysfunctions in adolescent rats exposed to repeated alcohol continue into adulthood) (Crews et al., 2000; Pascual et al., 2007).

Alcohol and other drugs of abuse induce changes in the Central Nervous System (CNS) that can lead to pathological behaviors related to addiction. In fact, the transition to alcoholism involves changes in the reinforcing and rewarding effects of alcohol use, a dysregulation of synaptic plasticity and the development of maladaptive stress responses (Kalivas and O'Brien, 2008; Koob, 2008). A variety of signals and neurotransmitters (e.g., dopamine, serotonin, glutamate, GABA, opioids, endocannabinoids…) in the CNS are implicated in the pathophysiology and development of alcoholism which play a prominent role in mediating behavioral and pharmacological effects of alcohol (Koob et al., 1998).

Over the past years, numerous studies have demonstrated the involvement of the endogenous cannabinoid system (ECS) in the behavioral and pharmacological effects of alcohol (Serrano and Parsons, 2011). The ECS includes endogenous ligands, cannabinoid receptors and the enzymatic machinery for the synthesis and inactivation of endocannabinoids. The main endocannabinoids are arachidonoylethanolamide (AEA) and 2-arachidonoylglycerol (2-AG), which bind to cannabinoid receptors to exert their effects. The cannabinoid receptors are G protein-coupled receptors and two types have been characterized and cloned, CB_1_ and CB_2_ (Howlett et al., 1990; Munro et al., 1993). Regarding the metabolic pathways for endocannabinoids, there are very complex enzymatic cascades for synthesis and inactivation that are crucial in regulating their levels. The primary pathway for AEA synthesis is mediated by a specific phospholipase D (NAPE-PLD) (Okamoto et al., 2004), while the synthetic pathway for 2-AG is mainly mediated by two sn-1-selective diacylglycerol lipases (DAGL-α and DAGL-β) (Bisogno et al., 2003). Finally, the inactivation of endocannabinoids is mediated by cellular reuptake and subsequent intracellular hydrolysis and both fatty acid amide hydrolase (FAAH) and monoacylglycerol lipase (MAGL) have been identified as enzymes primarily responsible for the degradation of AEA and 2-AG, respectively (Cravatt et al., 1996; Dinh et al., 2002).

The ECS has been widely studied in recent years due to its anti-inflammatory and homeostatic properties, which is of interest because alcohol abuse is associated with the induction of neuroinflammatory and neurodegenerative processes. In this regard, binge-like alcohol exposure increases the nuclear factor-*kappa* β (NF-κβ)-DNA binding activity, up-regulates the expression of cyclooxygenase-2 (COX-2), causes microglia activation (Knapp and Crews, 1999; Obernier et al., 2002a; Crews et al., 2006) and induces brain injury in the cortex and hippocampus associated with cognitive deficits (Crews et al., 2000; Obernier et al., 2002b; Tajuddin et al., 2014; Antón et al., 2016). However, it is important to consider that all the mentioned effects of alcohol use depend on several factors, including age of the subject, amount of alcohol consumed, duration and pattern of alcohol consumption (Kovacs and Messingham, 2002; Goral et al., 2008).

Long-term effects of alcohol exposure in animal models have revealed clear deleterious impact on emotional and cognitive processing, as well as on underlying inflammatory responses triggered by the unique ability of alcohol to direct activate natural immunity through toll-like receptor-4 (TLR4) (Fernandez-Lizarbe et al., 2009; Pascual et al., 2011). In fact, recent studies have reported the role of TLR4 in behavioral and cognitive dysfunctions using models of intermittent alcohol exposure in adolescence (Montesinos et al., 2015, 2016).

In this context, the present study aimed to characterize the impact of an adolescent intermittent alcohol exposure in emotional behaviors (open field and elevated-plus maze), cognitive responses (novel object recognition memory) and gene expression of primary signaling systems related to neuroinflammation, anxiety and stress in adult rats. The medial prefrontal cortex, amygdala, striatum and hippocampus were selected for gene expression analysis since they play a predominant role in alcohol-related behaviors and neuroadaptations.

## Materials and methods

### Animals and ethical statement

Twenty-four male Wistar rats (Charles River Laboratories, Barcelona, Spain) weighing 100–125 g on postnatal day (pnd) 24 were housed individually in a humidity and temperature-controlled vivarium on a 12 h light/dark cycle (lights off at 19:00 h). Rats were allowed to acclimatize to the new environment for 7 days before any experimental procedure was performed (pnd 31). Water and rat chow pellets were available *ad libitum*.

This study was carried out in accordance with the European Directive 2010/63/EU for the protection of animals used for scientific purposes and Spanish regulations (Real Decreto 53/2013 and 178/2004, Ley 32/2007 and 9/2003 and Decreto 320/2010) for the care and use of laboratory animals. The protocol was approved by the Ethic and research Committee at the Universidad de Malaga. All efforts were made to minimize animal suffering and social isolation, as well as to reduce the number of animals used.

### Experimental design

**Figure 1:**
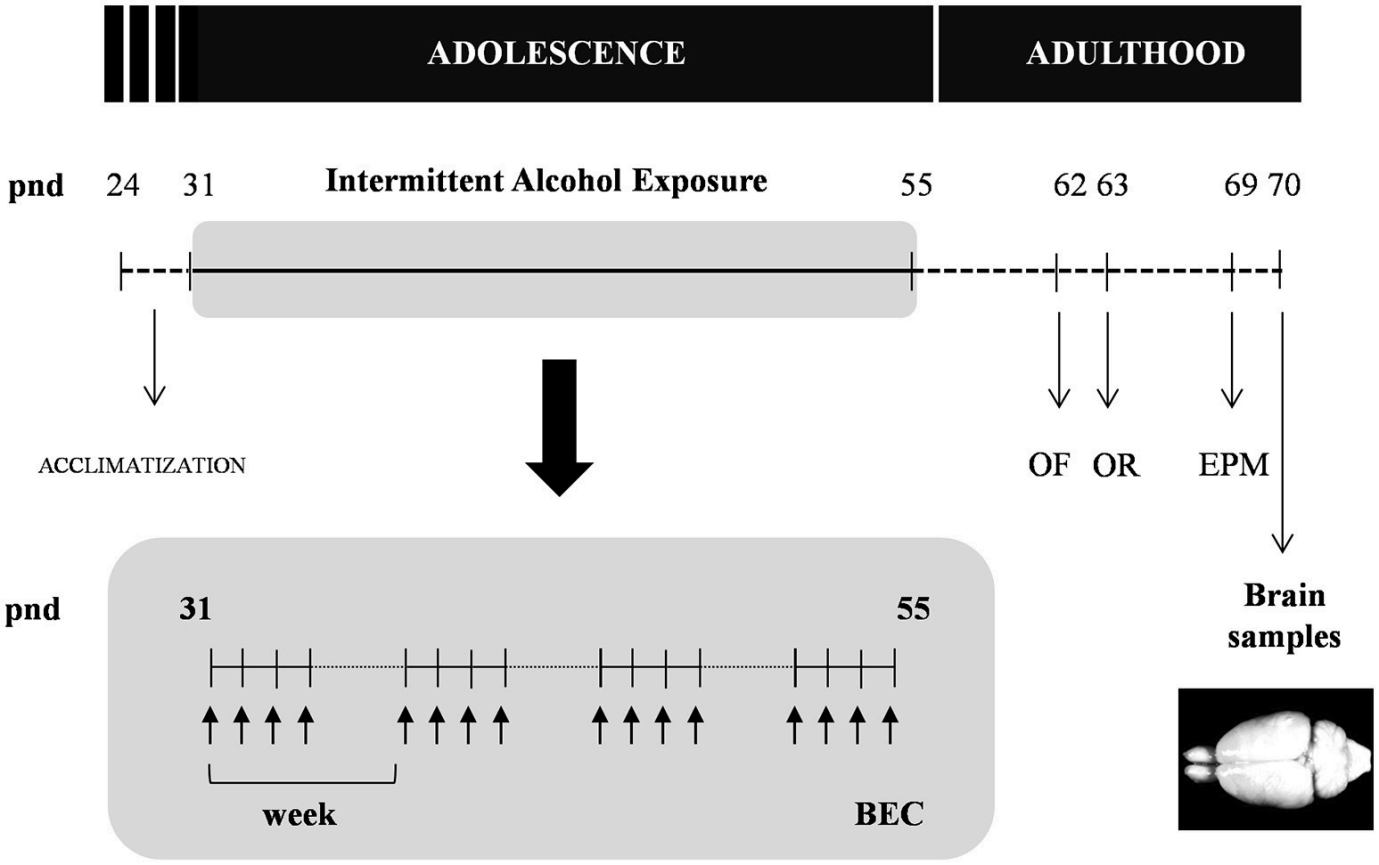
**Experimental design of the study**. Rats were exposed to an intermittent alcohol exposure for 4 weeks (from pnd 31 to 55). During this period, rats were randomly assigned to alcohol (repeated i.p. administration) or control (saline) group. Blood ethanol concentrations (BEC) were determined 60 min after the last alcohol exposure. The analyzes of behavior on the open field (OF), novel object recognition (OR) and elevated plus-maze (EPM) were carried out in the different groups at 1 and 2 weeks after the alcohol exposure. All animals were sacrificed 2 weeks after the last alcohol exposure and brain samples were collected.

As shown in Figure 1, adolescent rats (pnd 31) were randomly assigned to the experimental alcohol group following 4 weeks of intermittent alcohol exposure (*n* = 12) or control group (*n* = 12).

#### Intermittent alcohol procedure

An intraperitoneal (i.p.) administration of ethanol solution was used as intermittent intoxication during 4 weeks. Rats were injected weekly with 3 g/kg of ethanol (20% in saline, w/v) for 4 consecutive days followed by 3 days of alcohol deprivation. After the adolescent alcohol exposure, rats were left undisturbed in their home-cages before performing behavioral tests to evaluate locomotor activity, cognitive responses and anxiety-like behaviors. Following a schedule similar to the alcohol group, control animals received an injection of saline and they were also individually maintained in their home-cages for controlling the effects of social isolation on behavioral experiments and biochemical determinations previously reported in adolescence (Skelly et al., 2015).

### Determination of blood ethanol concentration

Rats were tail-bled 1 h after the last alcohol exposure. Blood was collected into a microtube containing anticoagulant (4 μl heparin; 1000 USP units/ml) and centrifuged at 2000 × g for 10 min. Serum was extracted and assayed for ethanol concentration (BEC) using the alcohol oxidase method with an AM1 Alcohol Analyzer (Analox Instrument, London, UK).

### Behavioral studies

Behavioral tests were conducted by trained observers who were unaware of the experimental conditions.

#### Open field test

Motor and anxiety-like behaviors were studied in an opaque open field (100 × 100 × 40 cm) divided into 16 squares with two zones; the center (4 squares) and periphery (12 squares) of the field. The open field was illuminated using a ceiling halogen lamp that was regulated to yield 350 lux at the center of the field. On the experimental day, the animals were placed in the center and the locomotor activity and time spent in the center and periphery were scored for 15 min. The number of crossings (expressed as number lines/squares crossed) and the percent of time spent in the center (time spent in the center/total time × 100) were calculated.

This task was also used as habituation session for the novel object recognition test.

#### Novel object recognition test

The novel object recognition procedure consists of habituation, familiarization, and test phases. Familiarization phase was conducted 24 h after the habituation phase. During the familiarization phase, animals were placed in the test field and allowed to explore for 3 min two identical objects. Then, animals were returned to their home-cage for 1 h. Test phase was conducted by placing rats for 3 min in the test field with 2 different objects, one was familiar and the other one was novel. Objects were made in plastic but different in shape, size and color. Between each test, the relative position of both objects were counterbalanced and permuted.

The time spent in exploring these objects were recorded in both phases. The exploration time was defined as the time that animals spent in licking, sniffing or touching each object, but it was not considered the time spent in standing or sitting on or leaning against each object (expressed in seconds). The novel object preference was determined using the discrimination ratio (expressed as the difference in time exploring the novel and the familiar objects/total exploration time).

#### Elevated plus-maze

The elevated plus-maze was made of opaque plastic and was composed of 2 oppositely positioned open arms (45 × 10 cm), 2 oppositely positioned closed arms of the same size and 50-cm-high walls. The arms were connected by a central and neutral area (10 × 10 cm). The entire apparatus was elevated 75 cm above a white floor and exposed to dim illumination (70 lux). At the beginning, rats were placed in the center of the maze, facing an open arm, and were allowed to freely explore the maze for 5 min. The number of entries (an arm entry was defined as all four paws in the arm zone) and the time spent in each arm were scored using a video monitor. The number of entries into the closed arms (expressed as number of entries into the closed arms) and the percent of time spent in the exposed arm (time spent in the open arms/total time × 100) were calculated.

### Sample collection

Two weeks after the last alcohol exposure (pnd 70), rats were anesthetized with sodium pentobarbital (50 mg/kg, i.p.) and brain samples were collected. The brains were quickly removed, immediately frozen on dry ice and stored at −80°C, until determination of the gene expression of proteins using quantitative real-time reverse transcription polymerase chain reaction (RT-qPCR).

### Dissection of the brain

The frozen brains were placed in acrylic rat brain matrices, and 2-mm thick slices were obtained using brain matrix razor blades. The medial prefrontal cortex (mPFC), striatum (accumbens, caudate, and putamen nucleus), entire amygdala and dorsal and ventral hippocampus were dissected out bilaterally and collected using a scalpel (mPFC and hippocampus) and a sample corer (striatum and amygdala). The localization of the brain regions was performed using a rat brain atlas and considering the bregma point as zero coordinate in the rostral-caudal coordinates: mPFC, +3.0 mm to +5.0 mm from bregma; striatum, +1.0 mm to −1.0 mm from bregma; amygdala, −1.0 mm to −3.0 mm from bregma; and hippocampus, −3.0 mm to −5.0 mm from bregma (Paxinos and Watson, 1998).

### RNA isolation and RT-qPCR analysis

Total RNA was extracted from brain samples using Trizol Reagent (Gibco BRL Life Technologies, Baltimore, MD, USA) and the concentrations were quantified using a spectrophotometer to ensure ratios of absorbance at 260 to 280 nm of 1.8–2.0. The reverse transcription was performed using the Transcriptor Reverse Transcriptase kit and random hexamer primers (Transcriptor RT; Roche Diagnostic, Mannheim, Germany). The RT-qPCR was performed using an ABI PRISM® 7300 Real-Time PCR System (Applied Biosystems, Foster City, CA, USA) and the FAM dye label format for the TaqMan Gene Expression Assays (Applied Biosystems). The absolute values from each sample were normalized relative to the housekeeping β-actin gene (*Actb*). The relative quantification was calculated using the ΔΔCt method and normalized to the control group. Primers for the RT-qPCR were obtained based on the Applied Biosystems genome database of rat mRNA references (http://bioinfo.appliedbiosystems.com/genome-database/gene-expression.html) (Table 1).

**Table 1:** **Primers references for TaqMan® Gene Expression Assays**.

**Gene symbol**	**Assay ID**	**GenBank accession number**	**Amplicon length**
***Actb***	Rn00667869_m1	NM_031144.3	91
***Cnr1***	Rn02758689_s1	NM_012784.4	92
***Cnr2***	Rn01637601_m1	NM_020543.4	68
***Dagla***	Rn01454304_m1	NM_001005886.1	67
***Daglb***	Rn01453771_m1	NM_001107120.1	98
***Faah***	Rn00577086_m1	NM_024132.3	63
***Mgll***	Rn00593297_m1	NM_138502.2	78
***Napepld***	Rn01786262_m1	NM_199381.1	71
***Ppara***	Rn00566193_m1	NM_013196.1	98
***Crh***	Rn01462137_m1	NM_031019.1	112
***Crhr1***	Rn00578611_m1	XM_006247542.2	58
***Crhr2***	Rn00575617_m1	NM_022714.1	82
***Npy***	Rn00561681_m1	NM_012614.2	63
***Npy1r***	Rn02769337_s1	NM_001113357.1	98
***Npy2r***	Rn00576733_s1	NM_023968.1	65
***Npy5r***	Rn02089867_s1	NM_012869.1	107
***Aif1***	Rn00574125_g1	NM_017196.3	126
***Gfap***	Rn01253033_m1	NM_017009.2	75
***Ptgs2***	Rn01483830_g1	NM_017232.3	69
***Rela***	Rn01502266_m1	NM_199267.2	67
***Tlr4***	Rn00569848_m1	NM_019178.1	127
***Tnf***	Rn99999017_m1	NM_012675.3	108

### Statistical analysis

All the data in the graphs are expressed as the means ± SEM. The statistical analysis was performed using GraphPad Prism version 5.04 (GraphPad Software, San Diego, CA, USA) and the normal distribution of data was evaluated by D'Agostino-Pearson omnibus test. Student's *t*-test and Welch's *t*-test for unequal variances were conducted to compare continuous variables between the two experimental groups (Control and Alcohol). Two-way analysis of variance (ANOVA) was conducted in the Novel Object Recognition Test using alcohol exposure (control and alcohol) and type of object (familiar and novel) as factors and using Bonferroni as *post hoc* test. Benjamini–Hochberg false discovery rate (FDR) approach was used for multiple comparisons (Benjamini et al., 2001) of the mRNA expression analysis. The statistics (*t*-statistic and *F*-statistic) and degrees of freedom were indicated in the description of results. The significance level was established at 0.05 and a *p*-value of less than 0.05 was considered statistically significant. Additionally, FDR adjusted significance levels were indicated for the multiple comparisons in each case.

## Results

In the present study, we examined adult rats exposed to intermittent alcohol exposures during adolescence. We assessed anxiety-like and cognitive behaviors as well as the mRNA expression of numerous enzymes and receptors involved in the ECS and other mediators linked to behavior, neuroinflammation and plasticity.

### Body weight gain and food intake during adolescent intermittent alcohol exposure

**Figure 2:**
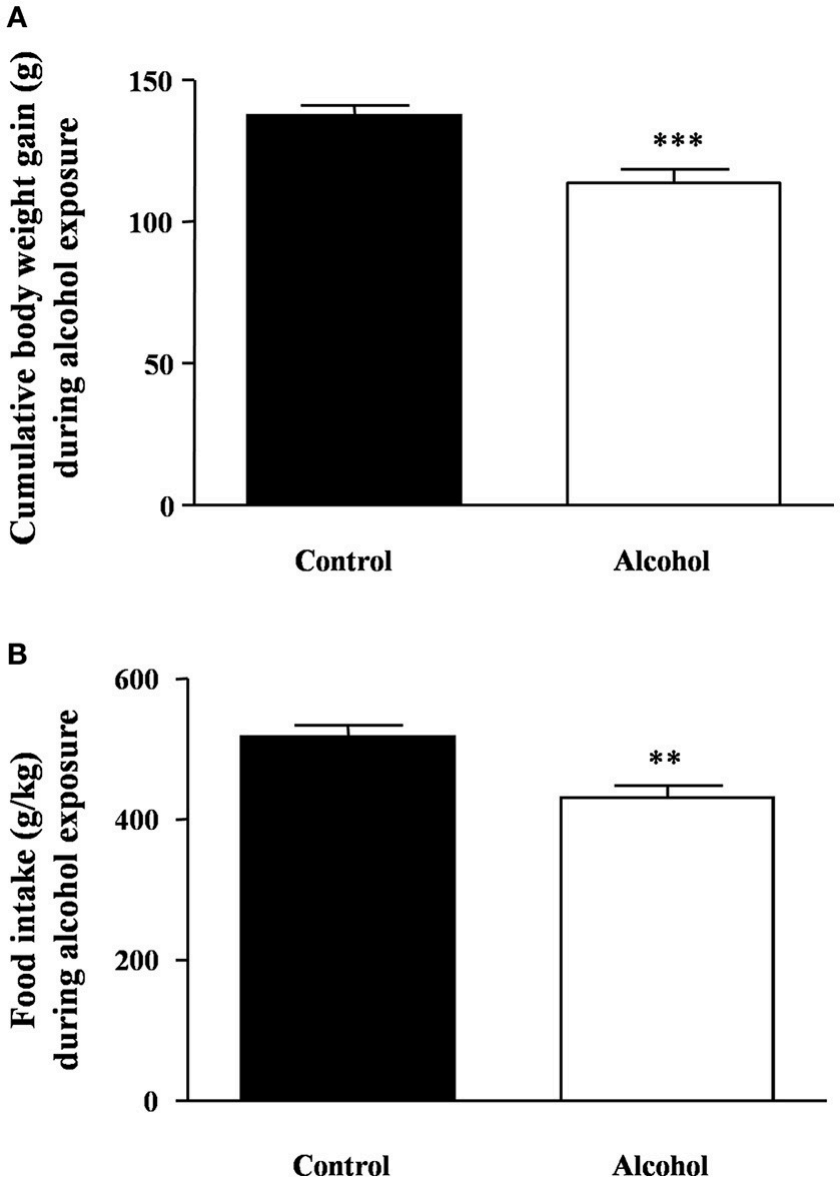
**Body weight gain and food intake during intermittent alcohol exposure in adolescent rats**. Cumulative body weight gain **(A)** and food intake **(B)** after 4 weeks of alcohol exposure. Bars represent the mean ± SEM (8–12 animals per group). Data were analyzed by Student's *t*-test (Welch's correction). ^**^p < 0.01 and ^***^p < 0.001 denote significant differences compared with the control group.

As shown in Figure 2A, during the period of alcohol exposure, there was a significant decrease in body weight gain in the alcohol group compared with the control group [*t*_(22)_ = 4.00, *p* < 0.001]. Similarly, the averages of food intake during these 4 weeks were also significantly different [*t*_(22)_ = 3.68, *p* = 0.001] (Figure 2B).

After the last alcohol exposure, the BEC in the alcohol group reached an average of 201 ± 6 mg/dl.

### Effects of intermittent alcohol exposure on locomotion and anxiety-like behaviors

We next explored whether alcohol exposure during adolescence induced locomotor or emotional alterations.

**Figure 3:**
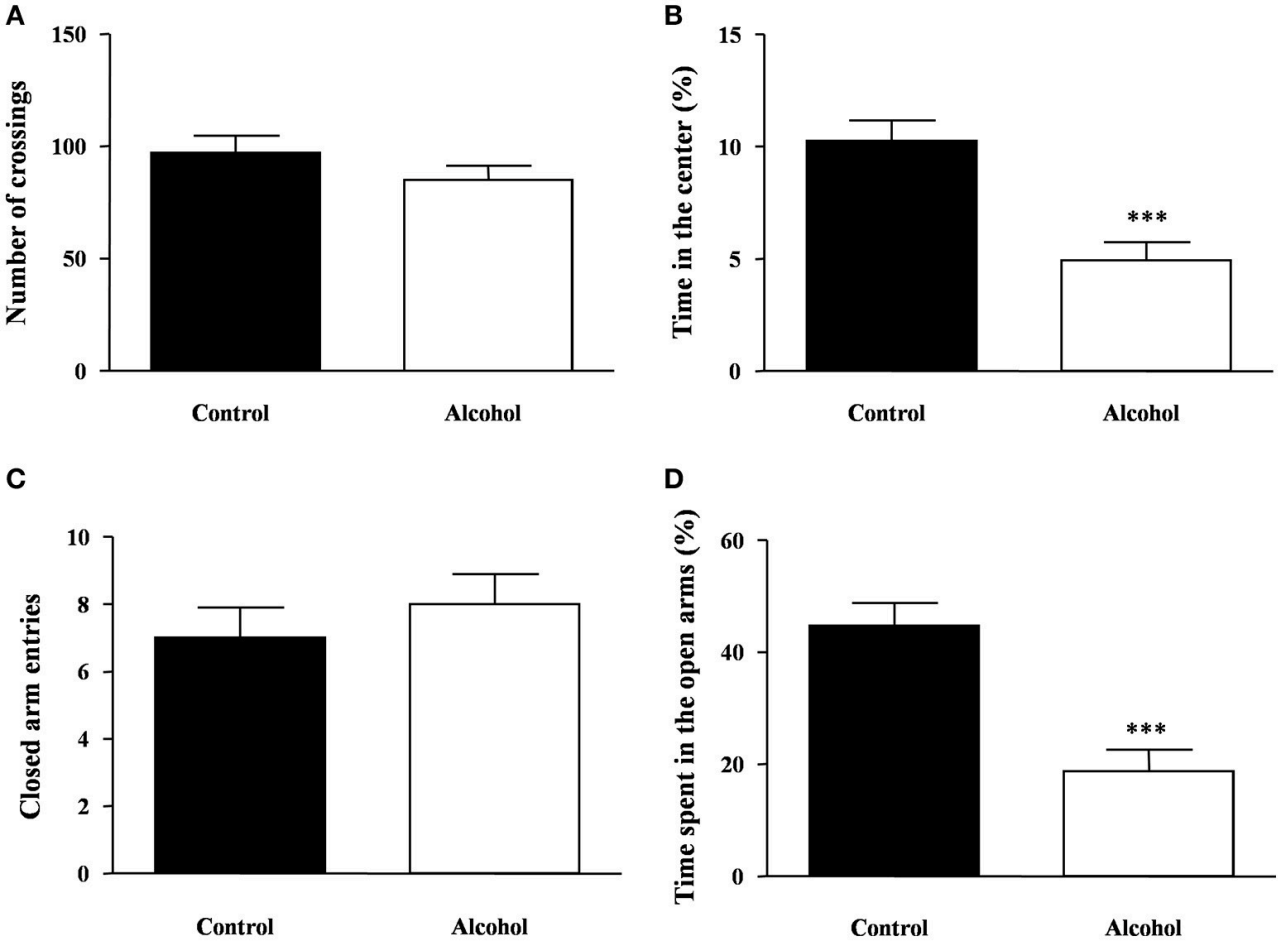
**Locomotion and anxiety-like behavior in adult rats exposed to intermittent adolescent alcohol**. Number of crossings **(A)** and time spent in the center of the field **(B)** were evaluated in the open field test 1 week after the last alcohol exposure (pnd 62). Closed arm entries **(C)** and time spent exploring the open arms **(D)** were evaluated in the elevated plus-maze 2 weeks after the last alcohol exposure (pnd 69). Bars represent the mean ± SEM (8–12 animals per group). Data were analyzed by Student's *t*-test (Welch's correction). ^***^p < 0.001 denotes significant differences compared with the control group.

One week after the last adolescent alcohol exposure, we measured spontaneous locomotor and exploratory activity using the open field test in rats. We observed no significant differences in the number of crossings between both groups (Figure 3A). Additionally, we measured anxiety-like behavior by time spent in the center area of the field (Figure 3B) and we detected a significant decrease in the time spent in the center area in the alcohol group compared with the control group [*t*_(14)_ = 4.19, *p* < 0.001].

The rats were also tested on the elevated plus-maze under novelty conditions 2 weeks after alcohol exposure. Regarding the locomotor activity in the elevated plus-maze, we found no differences in the number of entries in the closed arms between groups (Figure 3C). In this maze, we measured anxiety-like behavior by the time spent in the open arms and significant differences were found. As shown in Figure 3D, there was a significant decrease in the time of open arm exploration in the alcohol group compared with the control group [*t*_(14)_ = 4.65, *p* < 0.001].

### Effects of intermittent alcohol exposure on novel object recognition

**Figure 4:**
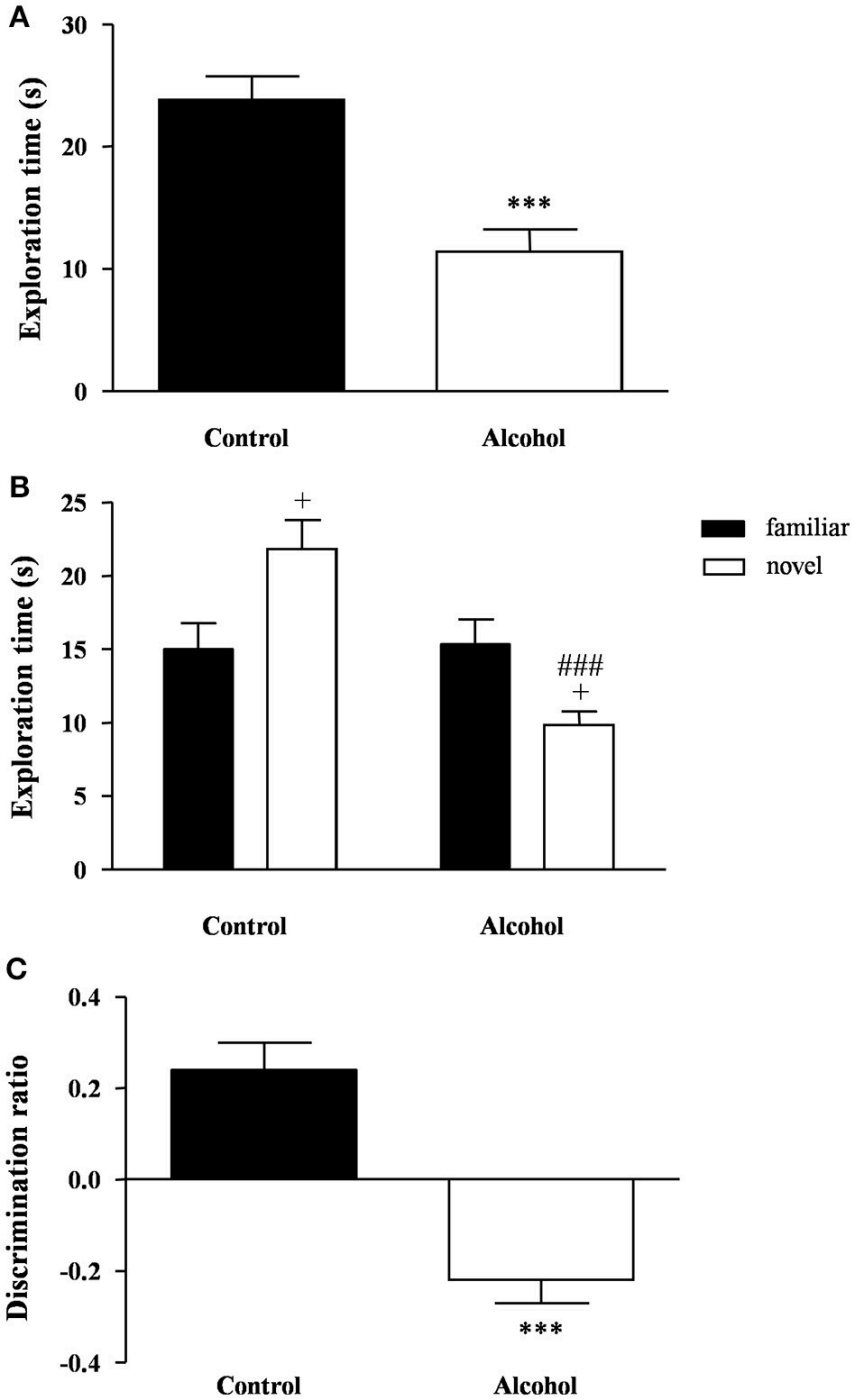
**Recognition memory in adult rats exposed to intermittent adolescent alcohol**. Exploration time **(A)** during the familiarization phase, exploration time for both familiar and novel object **(B)** during the test phase and discrimination ration **(C)** of the novel object. Bars represent the mean ± SEM (8–12 animals per group). Data were analyzed by Student's *t*-test (Welch's correction) and two-way ANOVA. ^***^p < 0.001 denotes significant differences compared with the control group. ^+^*p* < 0.05 denotes significant differences compared with time exploring the familiar object; and ^###^*p* < 0.001 denotes significant differences compared with time exploring the novel object in the control group.

One week after adolescent alcohol exposure, the recognition memory was measured using the novel object recognition task. During the familiarization phase, none of the groups displayed any preference for the objects but the statistical analysis revealed significant differences on the total exploration time (Figure 4A). Thus, the alcohol group spent less time to explore both objects than the control group [*t*_(21)_ = 4.73, *p* < 0.001].

One hour later, the test session was repeated using a novel object. A two-way ANOVA revealed a main effect of alcohol exposure on the exploration time [*F*_(1, 42)_ = 12.76, *p* < 0.001] but also an interaction between alcohol exposure and type of object [*F*_(1, 42)_ = 14.40, *p* < 0.001]. As shown in Figure 4B, the *post hoc* comparison indicated that the control group had a higher preference for the novel object relative to the familiar object (*p* < 0.05), whereas the alcohol group displayed a lower preference for the novel object (*p* < 0.05). Additionally, there was a significant decrease in the preference of the novel object in the alcohol group compared with the control group (*p* < 0.001).

Consequently, the alcohol group exhibited a negative discrimination ratio that indicated an impaired recognition memory [*t*_(21)_ = 5.83, *p* < 0.001] (Figure 4C).

### Effects of intermittent alcohol exposure on the gene expression of endocannabinoid system

We examined the effect of adolescent alcohol exposure on the gene expression encoding relevant enzymes and receptors involved in endocannabinoid signaling [receptors: CB_1_ (*Cnr1*), CB_2_ (*Cnr2*) and peroxisome proliferator-activated receptor-α (PPAR-α) (*Ppara*); synthesis enzymes: NAPE-PLD (*Napepld*), DAGL-α (*Dagla*) and DAGL-β (*Daglb*); degradation enzymes: FAAH (*Faah*) and MAGL (*Mgll*)] following 2 weeks after the last exposure.

#### Medial prefrontal cortex

**Figure 5:**
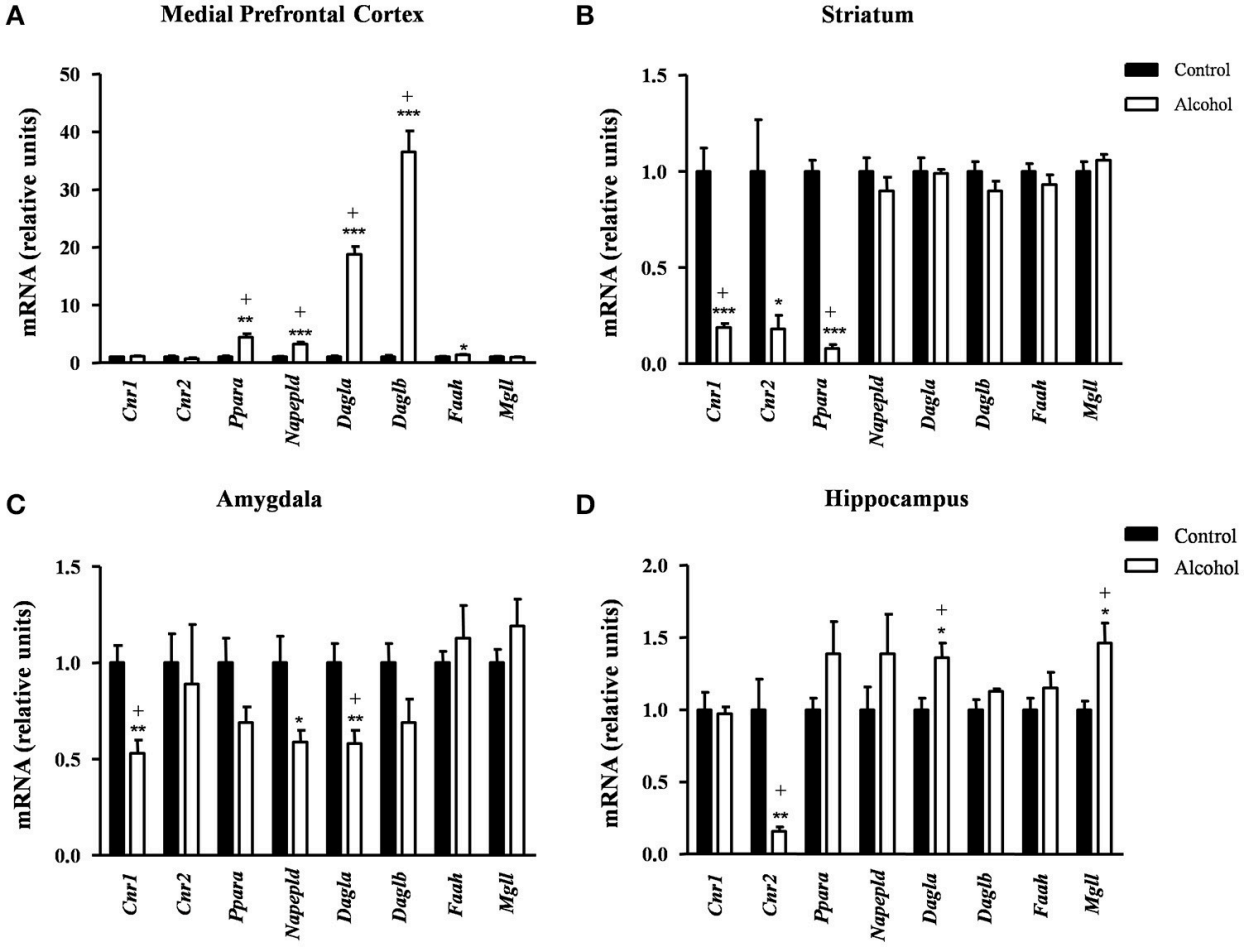
**Relative mRNA expression of endocannabinoid signaling-related genes in different brain regions of adult rats exposed to intermittent adolescent alcohol**. Relative mRNA expression of *Cnr1, Cnr2, Ppara, Napepld, Dagla, Daglb, Faah* and *Mgll* in the medial prefrontal cortex **(A)**, striatum **(B)**, amygdala **(C)**, and hippocampus **(D)**. Bars represent the mean ± SEM (8 animals per group). Data were analyzed by Student's *t*-test (Welch's correction) and Benjamini-Holchberg false discovery rate procedure for multiple comparisons. ^*^p < 0.05, ^**^p < 0.01 and ^***^p < 0.001 denote significant differences compared with the control group. (+) denotes significant differences compared with the control group after adjustment for multiple comparisons in each brain region.

In the mPFC (Figure 5A), the observed effects were mostly activatory. The mRNA expression of *Ppara* [*t*_(7)_ = 4.96, *p* = 0.002]; the synthesis enzymes *Napepld* [*t*_(14)_ = 6.12, *p* < 0.001], *Dagla* [*t*_(7)_ = 12.47, *p* < 0.001] and *Daglb* [*t*_(7)_ = 9.47, *p* < 0.001]; and *Faah* [*t*_(14)_ = 2.53, *p* = 0.024] were significantly increased in the alcohol group relative to the control group before adjustment for multiple comparisons. The FDR adjusted significance level (*q* = 0.031) indicated that all differences in the mRNA levels were still significant.

#### Striatum

Conversely, the adolescent exposure during adolescence produced only inhibitory effects, as shown in Figure 5B. Thus, rats exposed to alcohol displayed a significant decrease in the striatal mRNA expression of the receptors: *Cnr1*[*t*_(7)_ = 6.66, *p* < 0.001], *Cnr2* [*t*_(7)_ = 2.94, *p* = 0.022] and *Ppara* [*t*_(8)_ = 14.55, *p* < 0.001] compared with the control group. However, the FDR adjusted significance level (*q* = 0.013) showed a significant decrease in the expression of *Cnr1* and *Ppara*.

#### Amygdala

Similar to the striatum, we found again inhibitory effects in the amygdala (Figure 5C). The mRNA levels of *Cnr1* [*t*_(14)_ = 4.12, *p* = 0.001], *Napepld* [*t*_(9)_ = 2.69, *p* = 0.025] and *Dagla* [*t*_(14)_ = 3.44, *p* = 0.004] were significantly decreased in the alcohol group relative to the control group before adjustment for multiple comparisons. The subsequent correction (*q* = 0.013) revealed significant differences in the expression of *Cnr1* and *Dagla* but not in *Napepld*.

#### Hippocampus

In the hippocampus (Figure 5D), the alcohol group displayed a significant decrease in the mRNA levels of *Cnr2* [*t*_(7)_ = 3.96, *p* = 0.006] and a significant increase in both *Dagla* [*t*_(14)_ = 2.81, *p* = 0.014] and *Mgll* [*t*_(9)_ = 3.02, *p* = 0.015] relative to the control group. Consistently, the FDR adjusted significance level (*q* = 0.019) revealed that these differences in the mRNA expression were still significant.

Table S1 summarizes the adjustment for multiple comparisons of the mRNA expression of the ECS in each brain region.

### Effects of intermittent alcohol exposure during adolescence on the gene expression of neuropeptides linked to anxiety and stress

We also evaluated the effect of alcohol exposure during adolescence on the gene expression of neuropeptides linked to anxiety/stress responses and their receptors [neuropeptides: corticotropin-releasing factor (CRF) (*Crh*) and neuropeptides Y (NPY) (*Npy*); and receptors: CRF1R (*Crhr1*), CRF2R (*Crhr2*), NPY1R (*Npy1r*), NPY2R (*Npy2r*) and NPY5R (*Npy5r*)]. Determinations were conducted in the amygdala and hippocampus, two main regions belonging to the circuit that modulates emotional responses.

#### Amygdala

**Figure 6:**
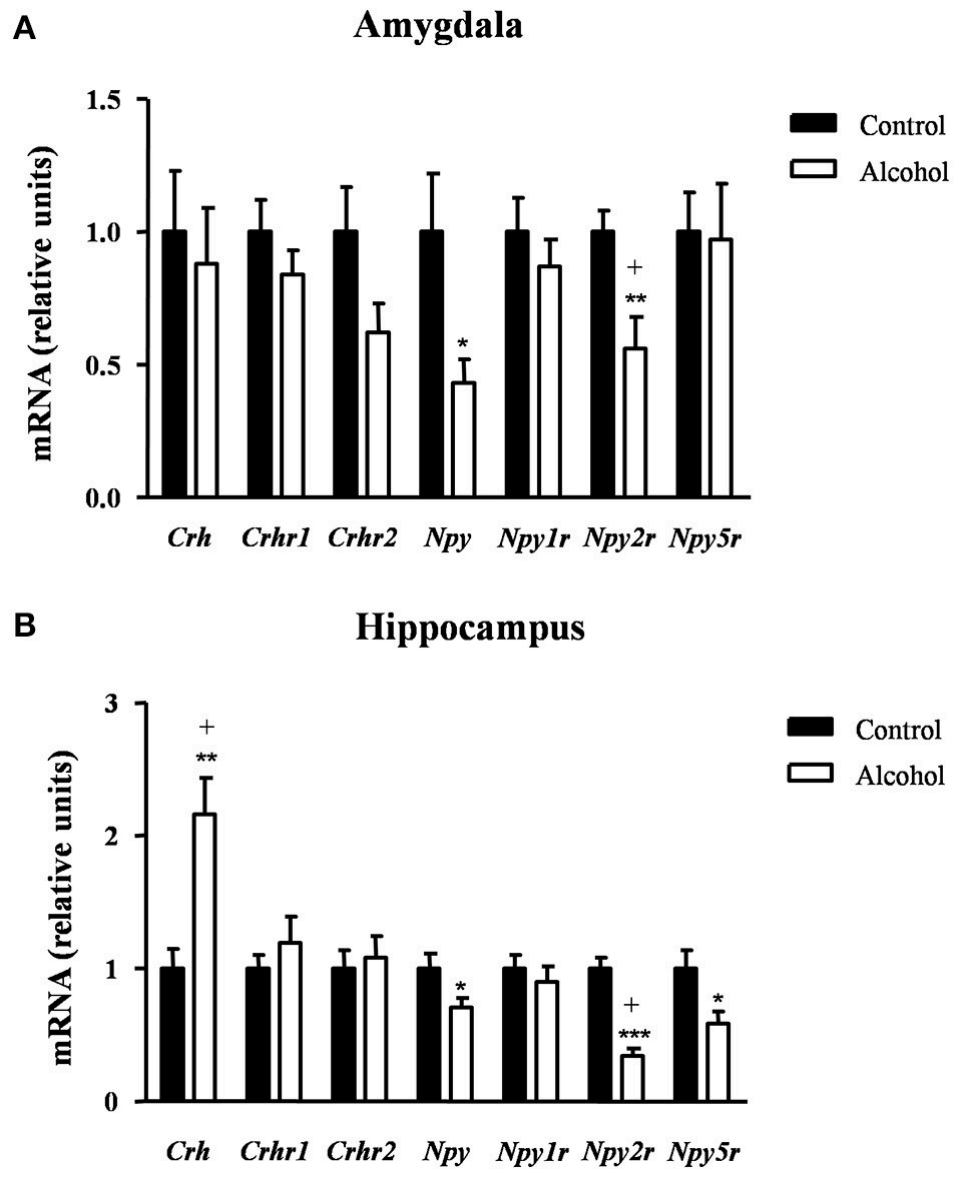
**Relative mRNA expression of markers of anxiety in different brain regions of adult rats exposed to intermittent adolescent alcohol**. Relative mRNA expression of *Crh, Crhr1, Crhr2, Npy, Npy1r, Npy2r*, and *Npy5r* in the amygdala **(A)** and hippocampus **(B)**. Bars represent the mean ± SEM (8 animals per group). Data were analyzed by Student's *t*-test (Welch's correction) and Benjamini-Holchberg false discovery rate procedure for multiple comparisons. ^*^p < 0.05, ^**^p < 0.01 and ^***^p < 0.001 denote significant differences compared with the control group. (+) denotes significant differences compared with the control group after adjustment for multiple comparisons in each brain region.

As shown in Figure 6A, the statistical analysis indicated no differences in the mRNA expression of *Crh* and its receptors but there were significant decreases in the expression of *Npy* [*t*_(9)_ = 2.40, *p* = 0.040] and *Npy2r* [*t*_(14)_ = 3.07, *p* = 0.007] in the alcohol group compared with the control group. However, the FDR adjusted significance level (*q* = 0.007) only remained significant differences between groups for *Npy2r*.

#### Hippocampus

In the hippocampus (Figure 6B), repeated *t*-tests showed significant differences in the mRNA levels of *Crh, Npy, Npy2r*, and *Npy5r* between groups. Thus, the alcohol group displayed higher *Crh* mRNA expression [*t*_(14)_ = 3.76, *p* = 0.002] and lower mRNA expression of *Npy* [*t*_(14)_ = 2.22, *p* = 0.043], *Npy2r* [*t*_(14)_ = 6.60, *p* < 0.001] and *Npy5r* [*t*_(14)_ = 2.46, *p* = 0.027] than the control group. After adjusting for multiple comparisons (*q* = 0.014) in the hippocampus, the significant differences were only observed in *Crh* and *Npy2r*.

Table S2 summarizes the adjustment for multiple comparisons of the mRNA expression of neuropeptides and receptors in the amygdala and hippocampus.

### Effects of intermittent alcohol exposure during adolescence on the gene expression of neuroinflammatory factors

Finally, we examined the effect of alcohol exposure during adolescence on the gene expression of relevant inflammatory-related markers [pro-inflammatory mediators: tumor necrosis factor-α (TNF-α) (*Tnf*), TLR4 (*Tlr4*), COX-2 (*Ptgs2*) and NF-κβ (*Rela*); and glia-activating factors: glial fibrillary acidic protein (GFAP) (*Gfap*) and allograft inflammatory factor-1 or microglia response factor (MRF-1) (*Aif1*)] following 2 weeks after the last alcohol exposure.

#### Medial prefrontal cortex

**Figure 7:**
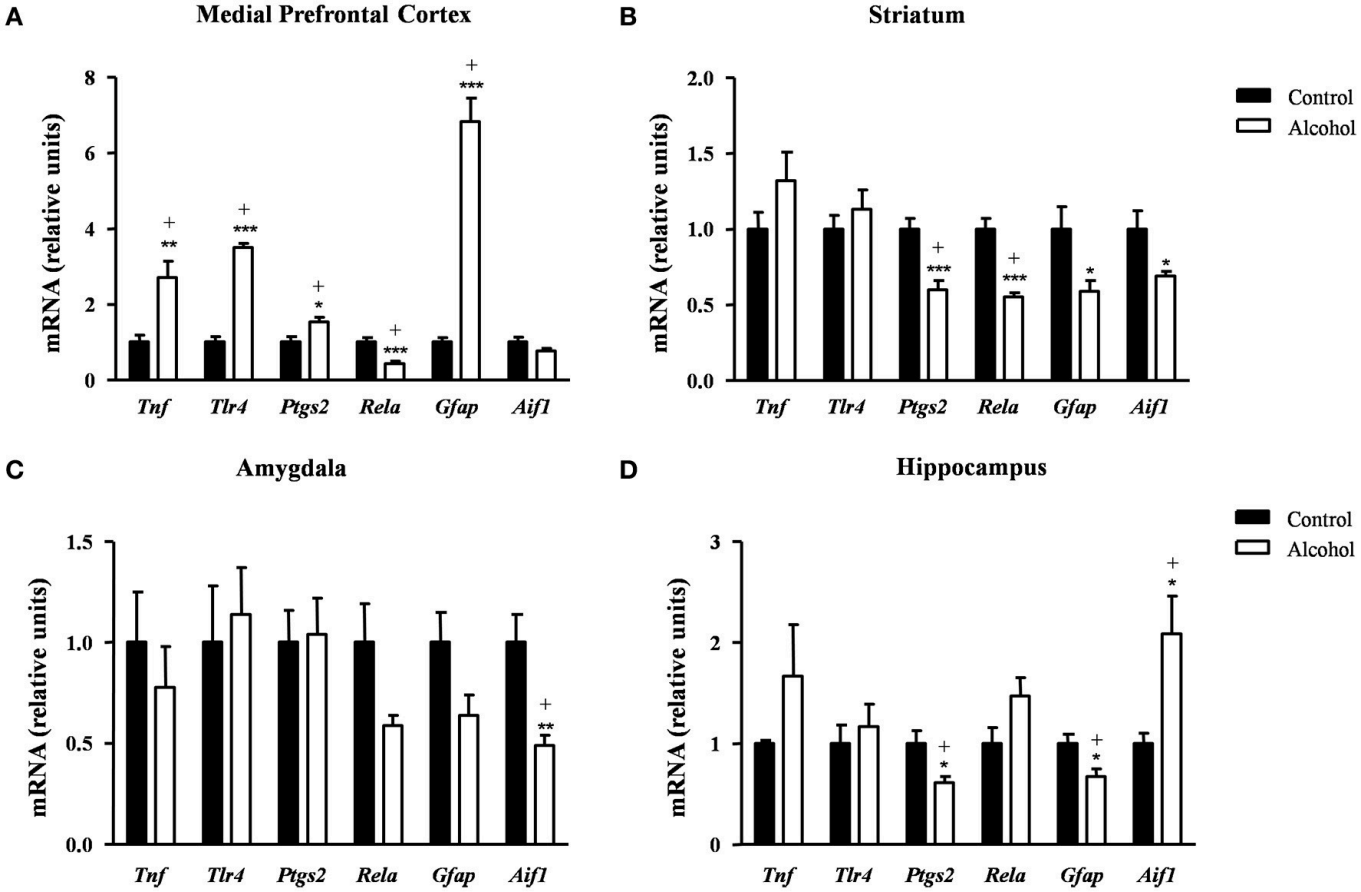
**Relative mRNA expression of neuroinflammatory factors in different brain regions of adult rats exposed to intermittent adolescent alcohol**. Relative mRNA expression of *Tnf, Tlr4, Ptgs2, Rela, Gfap* and *Aif1* mRNA in the medial prefrontal cortex **(A)**, striatum **(B)**, amygdala **(C)**, and hippocampus **(D)**. Bars represent the mean ± SEM (8 animals per group). Data were analyzed by Student's *t*-test (Welch's correction) and Benjamini–Holchberg false discovery rate procedure for multiple comparisons. ^*^p < 0.05, ^**^p < 0.01 and ^***^p < 0.001 denote significant differences compared with the control group. (+) denotes significant differences compared with the control group after adjustment for multiple comparisons in each brain region.

In the mPFC (Figure 7A), the mRNA levels of *Tnf* [*t*_(9)_ = 3.55, *p* = 0.006], *Tlr4* [*t*_(14)_ = 12.50, *p* < 0.001], *Ptgs2* [*t*_(14)_ = 2.67, *p* = 0.018] and *Gfap* [*t*_(7)_ = 9.08, *p* < 0.001] were significantly increased in the alcohol group in comparison with the control group. By contrast, the mRNA expression of *Rela* was significantly decreased in the alcohol group [*t*_(14)_ = 4.17, *p* < 0.001]. The adjustment for multiple comparisons (*q* = 0.042) confirmed all the significant differences found after repeated *t*-tests.

#### Striatum

In the striatum (Figure 7B), the statistical analysis showed a significant decrease in the mRNA levels of *Ptgs2* [*t*_(14)_ = 4.34, *p* < 0.001], *Rela* [*t*_(9)_ = 5.91, *p* < 0.001], *Gfap* [*t*_(14)_ = 2.48, *p* = 0.027] and *Aif1* [*t*_(7)_ = 2.51, *p* = 0.041] in the alcohol group compared with the control group. However, the FDR adjusted significance level (*q* = 0.018) remained significant differences only in the expression of *Ptgs2* and *Rela*.

#### Amygdala

As shown in Figure 7C, the alcohol group only displayed lower *Aif1* mRNA levels compared with the control group [*t*_(8)_ = 3.45, *p* = 0.008] and this difference was still observed after the correction for multiple comparisons (*q* = 0.008).

#### Hippocampus

Regarding the hippocampus (Figure 7D), the statistical analysis indicated that the alcohol group displayed lower mRNA levels of *Ptgs2* [*t*_(14)_ = 2.72, *p* = 0.017] and *Gfap* [*t*_(14)_ = 2.74, *p* = 0.016] and higher *Aif1* mRNA levels [*t*_(8)_ = 2.84, *p* = 0.022] compared with the control group. The adjustment for multiple comparisons (*q* = 0.025) confirmed the significance of all differences found using repeated *t*-tests.

Table S3 summarizes the adjustment for multiple comparisons of the mRNA expression of neuroinflammation-related factors in each brain region.

## Discussion

The present study in adult rats investigated the effects of intermittent alcohol intoxication on emotional behavior, cognition and the relative mRNA expression of genes from the endocannabinoid system (ECS), neuropeptides regulating anxiety- and alcohol-related behavior and mediators of neuroinflammation and plasticity. We must keep in mind that the social isolation of the animals in their home-cages throughout the experiments can affect the basal values in emotional behaviors (e.g., isolation induces anxiety-like behaviors and impairs fear extinction) and the expression of signaling systems in the brain (Skelly et al., 2015).

### Intermittent alcohol intoxication

As expected, the alcohol group showed elevated ethanol levels in the blood. These BECs were about 200 mg/dl and they are comparable to the concentrations reported in other studies conducted in adolescent Wistar rats using a model of binge-like drinking with i.p. injections of 3 g/kg, which reported 150–180 mg/dl 1 h postinjection (Przybycien-Szymanska et al., 2010, 2012). Although our procedure raised plasma alcohol levels in a binge-like manner [binge drinking is indeed defined as heavy episodic drinking within a BEC of 80 mg/dl or higher (NIAAA, 2004)], the alcohol administration was injected and it is possible that our model would yield higher alcohol doses than animals would consume voluntarily as it is expected in a binge drinking pattern (Spanagel, 2000). However, both patterns of excessive alcohol consumption are characterized by high BECs, and this can be critical in the effects of adolescent alcohol on behavior and expression of signaling systems in the brain.

During the alcohol exposure, we monitored the feeding behavior of adolescent rats by measuring body weight and food intake. Thus, the alcohol group displayed a lower body weight gain accompanied by a reduction in food intake during the alcohol exposure. These observations agree with previous studies reporting a significant decrease in body weight after i.p. administration of ethanol (Luz et al., 1996; Iwaniec and Turner, 2013). In fact, Lauing and colleagues reported decreased weight gain after modeling binge drinking using i.p. alcohol administration delivered on 2 consecutive days followed by 5 days of abstinence (Lauing et al., 2008). Several factors such as local inflammation and pain related to repeated injections, repeated acute ethanol intoxications with subsequent physical discomfort…could participate in this negative energy balance. However, there are studies reporting no differences in the final body weight after an intermittent binge-drinking exposure (Przybycien-Szymanska et al., 2010) although the total duration of this protocol was only 8 days.

### Effects on emotional behaviors and cognitive responses

After the alcohol exposure, locomotor activity, anxiety-like behavior and cognitive responses were assessed using a behavioral test battery consisting of the open field, elevated plus maze and novel object recognition task.

One week after the last alcohol session, we observed no differences in locomotor activity in the open field but there was an anxiogenic-like response to the test in the alcohol group compared with the control group, which could be related to the effects of alcohol deprivation on emotional behavior. These results are consistent with previous studies in rodents exposed to repeated binge-drinking during adolescence, which reported long-term anxiety disorders (Gilpin et al., 2012; Montesinos et al., 2016). One week later, the same rats were assessed in the elevated plus-maze and the anxiogenic-like behavior was also detected in the alcohol group. Ethanol withdrawal in rodents is associated with a negative affective state, including an enhanced anxiety-like behavior. Following a period of abstinence, rats spent significantly less time in the open arms of the elevated-plus maze than control animals (File et al., 1993; Moy et al., 1997; Wilson et al., 1998; Pandey et al., 2003). This behavioral profile can persist for as long as 28 days (Rasmussen et al., 2001). In our animals, the elevated anxiety in the alcohol group persisted unchanged for at least 2 weeks following the last alcohol exposure, suggesting enduring changes on emotionality.

A novel object recognition task was used to detect the existence of cognitive impairments because this paradigm relies on the innate preference that rodents display for exploring novel rather than familiar objects (Ennaceur and Delacour, 1988). To perform the task, we used a 1 h delay interval between the familiarization and novelty phases to consolidate a short-term memory that depends on the integrity of the hippocampal formation (García-Moreno et al., 2002). The results showed that rats exposed to alcohol displayed a decreased preference for the novel object over the familiar object but similar exploration time of the familiar object. This attracted our attention, because this less exploration time of the novel object was not associated with a locomotor impairment in this task, which was in agreement with the open field observations. In fact, the alcohol group displayed less exploration time during the familiarization phase when both identical objects were considered novel. Thus, the reduced preference for the novel object exhibited by the rats exposed to alcohol might be associated with the development of neophobia-induced anxiety and cognitive deficit, which is thought to prevent exploratory behavior (Myhrer, 1988).

### Effects on endocannabinoid system components

The brain is undergoing extensive maturation during adolescence. Similarly, the ECS is also undergoing maturational changes and some alteration of these processes of maturation can produces long-term alterations, including deficits in emotional behavior and cognition.

Several lines of evidence indicate that alcohol leads to neuroadaptations in endocannabinoid signaling mechanisms (Basavarajappa and Hungund, 1999; Basavarajappa et al., 2003). The present results showed that rats exposed to adolescent alcohol displayed alterations in the expression of endocannabinoid enzymes and receptors that appear to be reliant on the brain region, which is consistent with previous studies reporting regionally distinct effects on brain endocannabinoid levels after alcohol exposure (González et al., 2002, 2004; Rubio et al., 2007).

The mPFC is a key region for multiple cognitive functions, including executive function or anxiety. In addition, the ECS is involved in the regulation of emotional behaviors in this brain region (Rubino et al., 2008; McLaughlin et al., 2014; Morena et al., 2016). Thus, the negative impact of the alcohol to the adolescent mPFC may be associated to the observed alterations in cognition and behavior. We found that rats exposed to adolescent alcohol had a strong increase in the mRNA expression of *Napepld* and *Dagl* in this region. These observations suggest that the ECS is activated in response to the negative affective state associated to alcohol withdrawal. Additionally, the alcohol group displayed an increase in the mRNA expression of *Faah* and the receptor *Ppara*. It has been described that localized infusion of the FAAH inhibitor URB597 into the mPFC increases ethanol consumption by rats (Hansson et al., 2007). Also, a recent study has reported that pretreatment with an inhibitor of FAAH prevents against oxidative stress caused by binge ethanol consumption in the mPFC of adolescent rats (Pelição et al., 2016). Furthermore, PPAR-α is involved in the actions of other N-acylethanolamines with no endocannabinoid activity (e.g., oleoylethanolamide and palmitoylethanolamide) that have been reported to exhibit neuroprotective effects (Fu et al., 2003; Scuderi et al., 2012). Collectively these findings suggest a strengthening of the ECS that may reflect a homeostatic mechanism to prevent the neurotoxic effects induced by alcohol with a relevant role of other non-cannabinoid congeners in the alcohol exposure.

The ECS is an important modulator of neuroplasticity in the striatum. Overall, we observed that alcohol-exposed rats displayed a decreased mRNA expression of *Cnr1* and *Ppara*, as well as a decreasing trend in *Cnr2*. These results agree with preclinical studies that indicate a decrease in *Cnr1* mRNA levels after alcohol exposure in mice (Vinod et al., 2006) and rats (Adermark et al., 2011). Regarding the *Cnr2* mRNA levels, it is known that alcohol consumption alters the *Cnr2* expression in the brain and that an increased ethanol preference is associated with a reduced *Cnr2* expression in the striatum (Onaivi et al., 2008).

The amygdala is a crucial subcortical area that integrates reward, emotions, and conditioned learning. Previous studies have demonstrated drug-induced alterations in amygdalar endocannabinoid function (Orio et al., 2009; Kamprath et al., 2011; Schmidt et al., 2011). In the present study, we observed a decreasing trend in the receptors and synthesis enzymes but not in the degradative enzymes. Consistently, we have reported that chronic alcohol treatment alters the expression of different cannabinoid signaling-related genes in the amygdala (Serrano et al., 2012).

The hippocampus is a brain region involved in learning which cooperates with the amygdala to modulate emotion and belongs to the reward circuit. Numerous studies have shown that alcohol exposure alters hippocampal function (for review see Kutlu and Gould, 2016). Regarding the expression of the endocannabinoid signaling in the hippocampus, we found a dramatic decrease in the expression of *Cnr2*. In the hippocampus, such cannabinoid receptor is involved in the modulation of excitatory synapses (García-Gutiérrez et al., 2013; Kim and Li, 2015). A recent study has demonstrated a role of the hippocampal CB_2_ to modulate cognitive functions in mice (Li and Kim, 2016). Thus, the effects on *Cnr2* mRNA expression in our alcohol-exposed rats may be associated to the adverse effects of alcohol on synaptic plasticity (basically long-term potentiation and long-term depression processes) and the consequent defects in learning and memory (Lovinger and Roberto, 2013).

### Effects on neuropeptides linked to emotional behaviors

As mentioned above, the presence of anxiety is one of the most consistent features of alcohol withdrawal. Since the amygdala and hippocampus are key brain regions in the modulation of emotional behaviors and the ECS is also involved in the regulation of emotionality (Hill and Gorzalka, 2009), the present findings suggest that the differential changes observed in the ECS after adolescent alcohol exposure may be associated with different responses to anxiety. In this regard, to get a better understanding of the potential mechanisms underlying the anxiety effects that were observed, we analyzed the CRF and NPY signals in these regions. Furthermore, both neuropeptides are clearly involved in alcohol-related behaviors and binge alcohol drinking (Pleil et al., 2015).

Interestingly, we found an increase in the hippocampal expression of *Crh* mRNA in the alcohol group with no changes in the CRF receptors. In line with this, previous studies have associated CRF with binge drinking and alcohol dependence (Lowery et al., 2010; Gilpin et al., 2012; Pleil et al., 2015).

Regarding the NPY signal, in both brain areas the alcohol group displayed a down-regulation of this system with a strong decrease in the mRNA levels of *Npy2r* that may be associated with the anxiogenic-like behavior observed in this group. These findings are in agreement with a recent study showing that a decrease in the expression of receptors for NPY are associated with an increase in the anxiety-like behavior in adolescent rats exposed to repeated binge-like alcohol drinking (McClintick et al., 2016).

### Effects on neuroinflammatory signals

Since alcohol increases neuroinflammation through its ability of activating natural immunity, we evaluated the gene expression of factors involved in pro-inflammatory signaling pathways but also factors associated with plasticity and neurodevelopment.

The data indicate a region-specific susceptibility to the alcohol regulation of the mRNA expression of these factors, being these alterations more prominent in the mPFC. In line with this, binge drinking has been reported to produce alcohol-induced inflammatory PFC damage and this can be accompanied by reduced executive functions and compulsive behavior (Crews et al., 2000). Thus, we observed that adult rats exposed to adolescent alcohol displayed an overall increase in the mRNA expression of the neuroinflammation-related factors *Tlr4, Tnf* and *Ptgs2* in the mPFC. Several evidence indicates that alcohol induces the TLR4 signaling in the PFC (Vetreno and Crews, 2012; Pascual et al., 2014), triggering the induction of a cascade of pro-inflammatory mediators that affect, among others, cognitive impairments and anxiety-like behaviors associated with the alcohol abuse (Montesinos et al., 2015; Pascual et al., 2015).

TLR4 receptors are mediators of alcohol-induced inflammatory damage in the adolescent and adult brain (Alfonso-Loeches et al., 2010). In fact, Crews and colleagues have reported an increased *Tlr4* expression in the brain of alcoholics as well as in mice treated chronically with ethanol (Crews et al., 2013). In addition to TLR4, there was an up-regulation of *Tnf* and *Ptgs2* mRNA levels in alcohol-exposed rats, which is consistent with previous studies reporting that ethanol binge drinking is associated with increased cortical levels of TNF-α and COX-2 (Knapp and Crews, 1999; Antón et al., 2016). By contrast, the gene expression of various pro-inflammatory mediators were down-regulated (e.g., COX-2 mRNA levels were decreased in the striatum and hippocampus and NF-κβ mRNA levels were decreased in the mPFC and striatum), which appears to be incongruent with literature since alcohol exposure activates COX-2 and NF-κβ by triggering cytokine and chemokine release and neuroinflammation. In the mPFC, the decreased gene expression of *Rela* may be associated with the up-regulation of *Ppara* receptor observed in this brain region because it has been described that the activation of this nuclear receptor inhibits *Rela* (Stahel et al., 2008). Nevertheless, we have to keep in mind that changes in gene expression are often not concordant with protein expression (Vogel and Marcotte, 2012).

In addition to these factors and receptors, the inflammatory response is associated with astrocytes activation (Ransohoff and Brown, 2012). In fact, previous studies have demonstrated that *Gfap* levels are affected by alcohol exposure (Miguel-Hidalgo et al., 2006; Udomuksorn et al., 2011). Consistently, the gene expression of GFAP and MRF-1 were found up-regulated in the mPFC and hippocampus. Similar to COX-2 and NF-κβ, the gene expression of both astrocyte marker *Gfap* and microglial marker *Aif1* were also down-regulated in other brain regions (hippocampus and amygdala, respectively) and this was only reported in human alcoholic brain in conjunction with astrocytic loss (Lewohl et al., 2005).

### Summary

In summary, adult rats exposed to intermittent alcohol exposure during adolescence displayed anxiety-like behaviors and cognitive deficits related to recognition memory. Furthermore, the mRNA expression of some components of signaling systems involved in behavior, neuroinflammation and plasticity were found altered in these animals, with brain region-dependent changes.

Although adult rats exposed to adolescent alcohol had no alterations in locomotor activity, they exhibited a strong anxiogenic-like behavior. In addition to the anxiety-like response, we observed a clear cognitive deficit with a reduced preference for the novelty. These behavioral and cognitive effects in adulthood were accompanied by molecular changes in the brain.

Alterations in the mRNA expression of genes of the ECS appear to be reliant on the brain region. However, we observed an overall decreasing trend in the mRNA expression of the cannabinoid receptors in the alcohol group. This down-regulation can be associated to the adverse effects of alcohol on synaptic plasticity with cognitive consequences in adulthood such as deficits in learning and memory.

Whereas, the ECS is also associated with emotionality, neuropeptides such as CRF and NPY are clearly involved in alcohol-related behaviors and binge alcohol drinking. Thus, we observed changes in both signals that were linked to anxiogenic-like behaviors, in particular a decrease in the NPY signaling expression.

Regarding neuroinflammatory-related factors, our data indicated a brain region-specific susceptibility in the adult rats exposed to intermittent alcohol. These alterations were more prominent in the mPFC with an up-regulation of the mRNA levels of *Tlr4, Tnf, Ptgs2*, and *Gfap* and a decrease of *Rela*. The activation of PPAR-α inhibits NF-κβ and the mRNA expression of *Ppara* was increased in the mPFC, a key region for multiple cognitive functions. Interestingly, *Ppara* is involved in the anti-inflammatory and neuroprotective actions of endocannabinoid congeners. In contrast to the mPFC, the mRNA expression of *Ptgs2* and *Gfap* were down-regulated in the rest of brain regions.

### Limitations and perspectives

We are aware of the limitations of our study, mainly because of the exploratory nature of the present investigation and the necessity of performing future studies that include female subjects and alcohol exposure during adulthood. Furthermore, the protein expression and function of signaling systems and factors involved in behavior, neuroinflammation and neuroplasticity will open new lines of research using pharmacological and genetic approaches to characterize the association and regulation of these systems with emotional behavior and cognition in the context of adolescent alcohol and its long-term consequences.

The existence of sex differences in the response to alcohol and other drugs has been extensively reported (Becker and Koob, 2016) but we have conducted our experiments only in male rats for clarity. Studies using female rats will reveal whether the estrous cycle and gonadal hormones influence the behavioral and molecular alterations that we have observed here.

Although our data indicate changes in the mRNA expression of signaling systems in the brain and alterations in both emotional and cognitive responses of adult rats exposed to adolescent alcohol, we cannot link these molecular and behavioral effects exclusively to an alcohol exposure during adolescence because we have no rats exposed to alcohol during adulthood for comparison. Consequently, the specificity of the effects associated with intermittent alcohol exposure on these biological substrates will have to be elucidated.

Finally, additional studies will have to resolve the lack of the protein expression of these enzymes, factors and receptors because changes in gene expression level are frequently not reflected at the protein level. In fact, post-transcriptional, translational and degradation regulation must also be taken into account in the determination of protein concentrations because contribute at least as much as transcriptional itself (Vogel and Marcotte, 2012). Also, functional assays will be required to elucidate the role of these signaling systems in the appearance of emotional behaviors and addictive behaviors related to an adolescent alcohol exposure.

## Author contributions

All authors had full access to all data in the study and take responsibility for the integrity of the data and the accuracy of the data analyzes. Study concept and design: FR, AS. Acquisition of data: LS, FP, JD, JS, AG, EC. Analysis and interpretation of data: FP, FR, AS. Drafting of the manuscript: FR, AS. Critical revision of the manuscript for important intellectual content, obtained funding and study supervision: FP, FR, AS.

## Funding

The present study has been supported by the following institutions and grants: Instituto de Salud Carlos III (ISCIII) and European Union-European Regional Development Fund (EU-ERDF) (Subprograma RETICS Red de Trastornos Adictivos RD12/0028/0001); ISCIII and Ministerio de Economía y Competitividad (PI13/02261); Junta de Andalucía-Consejería de Igualdad, Salud y Políticas Sociales (PI-0823-2012 and PI-0228-2013); Ministerio de Sanidad, Servicios Sociales e Igualdad and Plan Nacional sobre Drogas (PNSD2015/047). FP (CP14/00212), JS (CP12/03109), and AS (CP14/00173) are recipients of a research contract from Miguel Servet Program of ISCIII and EU-ERDF.

### Conflict of interest statement

The authors declare that the research was conducted in the absence of any commercial or financial relationships that could be construed as a potential conflict of interest.

## References

[B1] Adermark L. Jonsson S. Ericson M. Söderpalm B. (2011). Intermittent ethanol consumption depresses endocannabinoid-signaling in the dorsolateral striatum of rat. Neuropharmacology 61, 1160–1165. 10.1016/j.neuropharm.2011.01.014 21251919

[B2] Alfonso-Loeches S. Pascual-Lucas M. Blanco A. M. Sanchez-Vera I. Guerri C. (2010). Pivotal role of TLR4 receptors in alcohol-induced neuroinflammation and brain damage. J. Neurosci. 30, 8285–8295. 10.1523/JNEUROSCI.0976-10.2010 20554880 PMC6634595

[B3] Antón M. Alén F. Gómez de Heras R. Serrano A. Pavón F. J. Leza J. C.. (2016). Oleoylethanolamide prevents neuroimmune HMGB1/TLR4/NF-kB danger signaling in rat frontal cortex and depressive-like behavior induced by ethanol binge administration. Addict. Biol. 10.1111/adb.12365. [Epub ahead of print]. 26857094

[B4] Basavarajappa B. S. Hungund B. L. (1999). Chronic ethanol increases the cannabinoid receptor agonist anandamide and its precursor N-arachidonoylphosphatidylethanolamine in SK-N-SH cells. J. Neurochem. 72, 522–528. 10.1046/j.1471-4159.1999.0720522.x 9930723

[B5] Basavarajappa B. S. Saito M. Cooper T. B. Hungund B. L. (2003). Chronic ethanol inhibits the anandamide transport and increases extracellular anandamide levels in cerebellar granule neurons. Eur. J. Pharmacol. 466, 73–83. 10.1016/S0014-2999(03)01557-7 12679143

[B6] Becker J. B. Koob G. F. (2016). Sex Differences in animal models: focus on addiction. Pharmacol. Rev. 68, 242–263. 10.1124/pr.115.011163 26772794 PMC4813426

[B7] Benjamini Y. Drai D. Elmer G. Kafkafi N. Golani I. (2001). Controlling the false discovery rate in behavior genetics research. Behav. Brain Res. 125, 279–284. 10.1016/S0166-4328(01)00297-2 11682119

[B8] Bisogno T. Howell F. Williams G. Minassi A. Cascio M. G. Ligresti A.. (2003). Cloning of the first sn1-DAG lipases points to the spatial and temporal regulation of endocannabinoid signaling in the brain. J. Cell Biol. 163, 463–468. 10.1083/jcb.200305129 14610053 PMC2173631

[B9] Cravatt B. F. Giang D. K. Mayfield S. P. Boger D. L. Lerner R. A. Gilula N. B. (1996). Molecular characterization of an enzyme that degrades neuromodulatory fatty-acid amides. Nature 384, 83–87. 10.1038/384083a0 8900284

[B10] Crews F. Nixon K. Kim D. Joseph J. Shukitt-Hale B. Qin L. (2006). BHT blocks NF-*k*B activation and ethanol-induced brain damage. Alcohol. Clin. Exp. Res. 30, 1938–1949. 10.1111/j.1530-0277.2006.00239.x 17067360

[B11] Crews F. T. Braun C. J. Hoplight B. Switzer R. C. III Knapp D. J. (2000). Binge ethanol consumption causes differential brain damage in young adolescent rats compared with adult rats. Alcohol. Clin. Exp. Res. 24, 1712–1723. 10.1111/j.1530-0277.2000.tb01973.x 11104119

[B12] Crews F. T. Qin L. Sheedy D. Vetreno R. P. Zou J. (2013). High mobility group box 1/Toll-like receptor danger signaling increases brain neuroimmune activation in alcohol dependence. Biol. Psychiatry 73, 602–612. 10.1016/j.biopsych.2012.09.030 23206318 PMC3602398

[B13] Dawson D. A. Goldstein R. B. Chou S. P. Ruan W. J. Grant B. F. (2008). Age at first drink and the first incidence of adult-onset DSM-IV alcohol use disorders. Alcohol. Clin. Exp. Res. 32, 2149–2160. 10.1111/j.1530-0277.2008.00806.x 18828796 PMC2760820

[B14] Dinh T. P. Carpenter D. Leslie F. M. Freund T. F. Katona I. Sensi S. L.. (2002). Brain monoglyceride lipase participating in endocannabinoid inactivation. Proc. Natl. Acad. Sci. U.S.A. 99, 10819–10824. 10.1073/pnas.152334899 12136125 PMC125056

[B15] Ennaceur A. Delacour J. (1988). A new one-trial test for neurobiological studies of memory in rats. 1: behavioral data. Behav. Brain Res. 31, 47–59. 10.1016/0166-4328(88)90157-X 3228475

[B16] Fernandez-Lizarbe S. Pascual M. Guerri C. (2009). Critical role of TLR4 response in the activation of microglia induced by ethanol. J. Immunol. 183, 4733–4744. 10.4049/jimmunol.0803590 19752239

[B17] File S. E. Andrews N. Al-Farhan M. (1993). Anxiogenic responses of rats on withdrawal from chronic ethanol treatment: effects of tianeptine. Alcohol. Alcohol. 28, 281–286. 8352839

[B18] Fu J. Gaetani S. Oveisi F. Lo Verme J. Serrano A. Rodriguez De Fonseca F.. (2003). Oleylethanolamide regulates feeding and body weight through activation of the nuclear receptor PPAR-alpha. Nature 425, 90–93. 10.1038/nature01921 12955147

[B19] García-Gutiérrez M. S. Ortega-Alvaro A. Busquets-Garcia A. Perez-Ortiz J. M. Caltana L. Ricatti M. J.. (2013). Synaptic plasticity alterations associated with memory impairment induced by deletion of CB2 cannabinoid receptors. Neuropharmacology 73, 388–396. 10.1016/j.neuropharm.2013.05.034 23796670

[B20] García-Moreno L. M. Conejo N. M. Capilla A. Garcia-Sanchez O. Senderek K. Arias J. L. (2002). Chronic ethanol intake and object recognition in young and adult rats. Prog. Neuropsychopharmacol. Biol. Psychiatry 26, 831–837. 10.1016/S0278-5846(01)00327-X 12369254

[B21] Gilpin N. W. Karanikas C. A. Richardson H. N. (2012). Adolescent binge drinking leads to changes in alcohol drinking, anxiety, and amygdalar corticotropin releasing factor cells in adulthood in male rats. PLoS ONE 7:e31466. 10.1371/journal.pone.0031466 22347484 PMC3275622

[B22] González S. Cascio M. G. Fernandez-Ruiz J. Fezza F. Di Marzo V. Ramos J. A. (2002). Changes in endocannabinoid contents in the brain of rats chronically exposed to nicotine, ethanol or cocaine. Brain Res. 954, 73–81. 10.1016/S0006-8993(02)03344-9 12393235

[B23] González S. Valenti M. De Miguel R. Fezza F. Fernandez-Ruiz J. Di Marzo V.. (2004). Changes in endocannabinoid contents in reward-related brain regions of alcohol-exposed rats, and their possible relevance to alcohol relapse. Br. J. Pharmacol. 143, 455–464. 10.1038/sj.bjp.0705963 15371286 PMC1575417

[B24] Goral J. Karavitis J. Kovacs E. J. (2008). Exposure-dependent effects of ethanol on the innate immune system. Alcohol 42, 237–247. 10.1016/j.alcohol.2008.02.003 18411007 PMC2453223

[B25] Guerri C. Bazinet A. Riley E. P. (2009). Foetal alcohol spectrum disorders and alterations in brain and behaviour. Alcohol Alcohol. 44, 108–114. 10.1093/alcalc/agn105 19147799 PMC2724862

[B26] Hansson A. C. Bermudez-Silva F. J. Malinen H. Hyytia P. Sanchez-Vera I. Rimondini R.. (2007). Genetic impairment of frontocortical endocannabinoid degradation and high alcohol preference. Neuropsychopharmacology 32, 117–126. 10.1038/sj.npp.1301034 16482090

[B27] Hill M. N. Gorzalka B. B. (2009). The endocannabinoid system and the treatment of mood and anxiety disorders. CNS Neurol. Disord. Drug Targets 8, 451–458. 10.2174/187152709789824624 19839936

[B28] Howlett A. C. Bidaut-Russell M. Devane W. A. Melvin L. S. Johnson M. R. Herkenham M. (1990). The cannabinoid receptor: biochemical, anatomical and behavioral characterization. Trends Neurosci. 13, 420–423. 10.1016/0166-2236(90)90124-S 1700516

[B29] Iwaniec U. T. Turner R. T. (2013). Intraperitoneal injection of ethanol results in drastic changes in bone metabolism not observed when ethanol is administered by oral gavage. Alcohol. Clin. Exp. Res. 37, 1271–1277. 10.1111/acer.12105 23550821 PMC3706497

[B30] Kalivas P. W. O'Brien C. (2008). Drug addiction as a pathology of staged neuroplasticity. Neuropsychopharmacology 33, 166–180. 10.1038/sj.npp.1301564 17805308

[B31] Kamprath K. Romo-Parra H. Haring M. Gaburro S. Doengi M. Lutz B.. (2011). Short-term adaptation of conditioned fear responses through endocannabinoid signaling in the central amygdala. Neuropsychopharmacology 36, 652–663. 10.1038/npp.2010.196 20980994 PMC3055679

[B32] Kim J. Li Y. (2015). Chronic activation of CB2 cannabinoid receptors in the hippocampus increases excitatory synaptic transmission. J. Physiol. 593, 871–886. 10.1113/jphysiol.2014.286633 25504573 PMC4398527

[B33] Knapp D. J. Crews F. T. (1999). Induction of cyclooxygenase-2 in brain during acute and chronic ethanol treatment and ethanol withdrawal. Alcohol. Clin. Exp. Res. 23, 633–643. 10.1111/j.1530-0277.1999.tb04165.x 10235299

[B34] Koob G. F. (2008). A role for brain stress systems in addiction. Neuron 59, 11–34. 10.1016/j.neuron.2008.06.012 18614026 PMC2748830

[B35] Koob G. F. Roberts A. J. Schulteis G. Parsons L. H. Heyser C. J. Hyytia P.. (1998). Neurocircuitry targets in ethanol reward and dependence. Alcohol. Clin. Exp. Res. 22, 3–9. 10.1111/j.1530-0277.1998.tb03611.x 9514280

[B36] Kovacs E. J. Messingham K. A. (2002). Influence of alcohol and gender on immune response. Alcohol Res. Health 26, 257–263. 12875035 PMC6676685

[B37] Kutlu M. G. Gould T. J. (2016). Effects of drugs of abuse on hippocampal plasticity and hippocampus-dependent learning and memory: contributions to development and maintenance of addiction. Learn. Mem. 23, 515–533. 10.1101/lm.042192.116 27634143 PMC5026208

[B38] Lauing K. Himes R. Rachwalski M. Strotman P. Callaci J. J. (2008). Binge alcohol treatment of adolescent rats followed by alcohol abstinence is associated with site-specific differences in bone loss and incomplete recovery of bone mass and strength. Alcohol 42, 649–656. 10.1016/j.alcohol.2008.08.005 19038696 PMC2633927

[B39] Lewohl J. M. Wixey J. Harper C. G. Dodd P. R. (2005). Expression of MBP, PLP, MAG, CNP, and GFAP in the human alcoholic brain. Alcohol. Clin. Exp. Res. 29, 1698–1705. 10.1097/01.alc.0000179406.98868.59 16205370

[B40] Li Y. Kim J. (2016). CB2 cannabinoid receptor knockout in mice impairs contextual long-term memory and enhances spatial working memory. Neural Plast. 2016:9817089. 10.1155/2016/9817089 26819779 PMC4706977

[B41] Lovinger D. M. Roberto M. (2013). Synaptic effects induced by alcohol. Curr. Top. Behav. Neurosci. 13, 31–86. 10.1007/978-3-642-28720-6_143 21786203 PMC4791588

[B42] Lowery E. G. Spanos M. Navarro M. Lyons A. M. Hodge C. W. Thiele T. E. (2010). CRF-1 antagonist and CRF-2 agonist decrease binge-like ethanol drinking in C57BL/6J mice independent of the HPA axis. Neuropsychopharmacology 35, 1241–1252. 10.1038/npp.2009.209 20130533 PMC2927867

[B43] Luz J. Griggio M. A. Plapler H. De-Meo-Bancher M. Carvalho-Kosmiskas J. V. (1996). Effects of ethanol on energy balance of rats and the inappropriateness of intraperitoneal injection. Alcohol 13, 575–580. 10.1016/S0741-8329(96)00070-5 8949952

[B44] McClintick J. N. McBride W. J. Bell R. L. Ding Z. M. Liu Y. Xuei X.. (2016). Gene expression changes in glutamate and GABA-A receptors, neuropeptides, ion channels, and cholesterol synthesis in the periaqueductal gray following binge-like alcohol drinking by adolescent alcohol-preferring (P) rats. Alcohol. Clin. Exp. Res. 40, 955–968. 10.1111/acer.13056 27061086 PMC4844794

[B45] McLaughlin R. J. Hill M. N. Gorzalka B. B. (2014). A critical role for prefrontocortical endocannabinoid signaling in the regulation of stress and emotional behavior. Neurosci. Biobehav. Rev. 42, 116–131. 10.1016/j.neubiorev.2014.02.006 24582908

[B46] Miguel-Hidalgo J. J. Overholser J. C. Meltzer H. Y. Stockmeier C. A. Rajkowska G. (2006). Reduced glial and neuronal packing density in the orbitofrontal cortex in alcohol dependence and its relationship with suicide and duration of alcohol dependence. Alcohol. Clin. Exp. Res. 30, 1845–1855. 10.1111/j.1530-0277.2006.00221.x 17067348 PMC2921167

[B47] Montesinos J. Pascual M. Pla A. Maldonado C. Rodriguez-Arias M. Minarro J.. (2015). TLR4 elimination prevents synaptic and myelin alterations and long-term cognitive dysfunctions in adolescent mice with intermittent ethanol treatment. Brain Behav. Immun. 45, 233–244. 10.1016/j.bbi.2014.11.015 25486089

[B48] Montesinos J. Pascual M. Rodriguez-Arias M. Minarro J. Guerri C. (2016). Involvement of TLR4 in the long-term epigenetic changes, rewarding and anxiety effects induced by intermittent ethanol treatment in adolescence. Brain Behav. Immun. 53, 159–171. 10.1016/j.bbi.2015.12.006 26686767

[B49] Morena M. Patel S. Bains J. S. Hill M. N. (2016). Neurobiological interactions between stress and the endocannabinoid system. Neuropsychopharmacology 41, 80–102. 10.1038/npp.2015.166 26068727 PMC4677118

[B50] Moy S. S. Knapp D. J. Criswell H. E. Breese G. R. (1997). Flumazenil blockade of anxiety following ethanol withdrawal in rats. Psychopharmacology (Berl). 131, 354–360. 10.1007/s002130050303 9226737

[B51] Munro S. Thomas K. L. Abu-Shaar M. (1993). Molecular characterization of a peripheral receptor for cannabinoids. Nature 365, 61–65. 10.1038/365061a0 7689702

[B52] Myhrer T. (1988). Exploratory behavior and reaction to novelty in rats with hippocampal perforant path systems disrupted. Behav. Neurosci. 102, 356–362. 10.1037/0735-7044.102.3.356 3395447

[B53] Nagel B. J. Schweinsburg A. D. Phan V. Tapert S. F. (2005). Reduced hippocampal volume among adolescents with alcohol use disorders without psychiatric comorbidity. Psychiatry Res. 139, 181–190. 10.1016/j.pscychresns.2005.05.008 16054344 PMC2270700

[B54] NIAAA (2004). NIAAA Council Approves Definition of Binge Drinking. Bethesda: National Institutes of Health NIAAA Newsletter.

[B55] Obernier J. A. Bouldin T. W. Crews F. T. (2002a). Binge ethanol exposure in adult rats causes necrotic cell death. Alcohol. Clin. Exp. Res. 26, 547–557. 10.1111/j.1530-0277.2002.tb02573.x 11981132

[B56] Obernier J. A. White A. M. Swartzwelder H. S. Crews F. T. (2002b). Cognitive deficits and CNS damage after a 4-day binge ethanol exposure in rats. Pharmacol. Biochem. Behav. 72, 521–532. 10.1016/S0091-3057(02)00715-3 12175448

[B57] Oesterle S. Hill K. G. Hawkins J. D. Guo J. Catalano R. F. Abbott R. D. (2004). Adolescent heavy episodic drinking trajectories and health in young adulthood. J. Stud. Alcohol. 65, 204–212. 10.15288/jsa.2004.65.204 15151351 PMC1876676

[B58] Okamoto Y. Morishita J. Tsuboi K. Tonai T. Ueda N. (2004). Molecular characterization of a phospholipase D generating anandamide and its congeners. J. Biol. Chem. 279, 5298–5305. 10.1074/jbc.M306642200 14634025

[B59] Onaivi E. S. Ishiguro H. Gong J. P. Patel S. Meozzi P. A. Myers L.. (2008). Functional expression of brain neuronal CB2 cannabinoid receptors are involved in the effects of drugs of abuse and in depression. Ann. N.Y Acad. Sci. 1139, 434–449. 10.1196/annals.1432.036 18991891 PMC3922202

[B60] Orio L. Edwards S. George O. Parsons L. H. Koob G. F. (2009). A role for the endocannabinoid system in the increased motivation for cocaine in extended-access conditions. J. Neurosci. 29, 4846–4857. 10.1523/JNEUROSCI.0563-09.2009 19369553 PMC2688678

[B61] Pandey S. C. Roy A. Zhang H. (2003). The decreased phosphorylation of cyclic adenosine monophosphate (cAMP) response element binding (CREB) protein in the central amygdala acts as a molecular substrate for anxiety related to ethanol withdrawal in rats. Alcohol. Clin. Exp. Res. 27, 396–409. 10.1097/01.ALC.0000056616.81971.49 12658105

[B62] Pascual M. Balino P. Alfonso-Loeches S. Aragon C. M. Guerri C. (2011). Impact of TLR4 on behavioral and cognitive dysfunctions associated with alcohol-induced neuroinflammatory damage. Brain Behav Immun. 25(Suppl. 1), S80–S91. 10.1016/j.bbi.2011.02.012 21352907

[B63] Pascual M. Balino P. Aragon C. M. Guerri C. (2015). Cytokines and chemokines as biomarkers of ethanol-induced neuroinflammation and anxiety-related behavior: role of TLR4 and TLR2. Neuropharmacology 89, 352–359. 10.1016/j.neuropharm.2014.10.014 25446779

[B64] Pascual M. Blanco A. M. Cauli O. Minarro J. Guerri C. (2007). Intermittent ethanol exposure induces inflammatory brain damage and causes long-term behavioural alterations in adolescent rats. Eur. J. Neurosci. 25, 541–550. 10.1111/j.1460-9568.2006.05298.x 17284196

[B65] Pascual M. Pla A. Minarro J. Guerri C. (2014). Neuroimmune activation and myelin changes in adolescent rats exposed to high-dose alcohol and associated cognitive dysfunction: a review with reference to human adolescent drinking. Alcohol. Alcohol. 49, 187–192. 10.1093/alcalc/agt164 24217958

[B66] Paxinos G. Watson C. (1998). The Rat Brain in Stereotaxic Coordinates. New York, NY: Academic Press; Spiral Bound.

[B67] Pelição R. Santos M. C. Freitas-Lima L. C. Meyrelles S. S. Vasquez E. C. Nakamura-Palacios E. M.. (2016). URB597 inhibits oxidative stress induced by alcohol binging in the prefrontal cortex of adolescent rats. Neurosci. Lett. 624, 17–22. 10.1016/j.neulet.2016.04.068 27150075

[B68] Pleil K. E. Rinker J. A. Lowery-Gionta E. G. Mazzone C. M. McCall N. M. Kendra A. M.. (2015). NPY signaling inhibits extended amygdala CRF neurons to suppress binge alcohol drinking. Nat. Neurosci. 18, 545–552. 10.1038/nn.3972 25751534 PMC4376619

[B69] Przybycien-Szymanska M. M. Gillespie R. A. Pak T. R. (2012). 17beta-Estradiol is required for the sexually dimorphic effects of repeated binge-pattern alcohol exposure on the HPA axis during adolescence. PLoS ONE 7:e32263. 10.1371/journal.pone.0032263 22384198 PMC3284554

[B70] Przybycien-Szymanska M. M. Rao Y. S. Pak T. R. (2010). Binge-pattern alcohol exposure during puberty induces sexually dimorphic changes in genes regulating the HPA axis. Am. J. Physiol. Endocrinol. Metab. 298, E320–E328. 10.1152/ajpendo.00615.2009 19952347 PMC2822472

[B71] Ransohoff R. M. Brown M. A. (2012). Innate immunity in the central nervous system. J. Clin. Invest. 122, 1164–1171. 10.1172/JCI58644 22466658 PMC3314450

[B72] Rasmussen D. D. Mitton D. R. Green J. Puchalski S. (2001). Chronic daily ethanol and withdrawal: 2. Behavioral changes during prolonged abstinence. Alcohol. Clin. Exp. Res. 25, 999–1005. 10.1111/j.1530-0277.2001.tb02308.x 11505024

[B73] Read J. P. Beattie M. Chamberlain R. Merrill J. E. (2008). Beyond the “Binge” threshold: heavy drinking patterns and their association with alcohol involvement indices in college students. Addict. Behav. 33, 225–234. 10.1016/j.addbeh.2007.09.001 17997047

[B74] Rubino T. Realini N. Castiglioni C. Guidali C. Vigano D. Marras E.. (2008). Role in anxiety behavior of the endocannabinoid system in the prefrontal cortex. Cereb. Cortex 18, 1292–1301. 10.1093/cercor/bhm161 17921459

[B75] Rubio M. Mchugh D. Fernandez-Ruiz J. Bradshaw H. Walker J. M. (2007). Short-term exposure to alcohol in rats affects brain levels of anandamide, other N-acylethanolamines and 2-arachidonoyl-glycerol. Neurosci. Lett. 421, 270–274. 10.1016/j.neulet.2007.05.052 17574742 PMC2966860

[B76] Schmidt K. Krishnan B. Xia Y. Sun A. Orozco-Cabal L. Pollandt S.. (2011). Cocaine withdrawal reduces group I mGluR-mediated long-term potentiation via decreased GABAergic transmission in the amygdala. Eur. J. Neurosci. 34, 177–189. 10.1111/j.1460-9568.2011.07769.x 21749491 PMC3138813

[B77] Scuderi C. Valenza M. Stecca C. Esposito G. Carratu M. R. Steardo L. (2012). Palmitoylethanolamide exerts neuroprotective effects in mixed neuroglial cultures and organotypic hippocampal slices via peroxisome proliferator-activated receptor-alpha. J. Neuroinflammation 9:49 10.1186/1742-2094-9-49 22405189 PMC3315437

[B78] Serrano A. Parsons L. H. (2011). Endocannabinoid influence in drug reinforcement, dependence and addiction-related behaviors. Pharmacol. Ther. 132, 215–241. 10.1016/j.pharmthera.2011.06.005 21798285 PMC3209522

[B79] Serrano A. Rivera P. Pavon F. J. Decara J. Suárez J. Rodriguez de Fonseca F.. (2012). Differential effects of single versus repeated alcohol withdrawal on the expression of endocannabinoid system-related genes in the rat amygdala. Alcohol Clin. Exp. Res. 36, 984–894. 10.1111/j.1530-0277.2011.01686.x 22141465 PMC3297719

[B80] Skelly M. J. Chappell A. E. Carter E. Weiner J. L. (2015). Adolescent social isolation increases anxiety-like behavior and ethanol intake and impairs fear extinction in adulthood: possible role of disrupted noradrenergic signaling. Neuropharmacology 97, 149–159. 10.1016/j.neuropharm.2015.05.025 26044636 PMC4537360

[B81] Spanagel R. (2000). Recent animal models of alcoholism. Alcohol Res. Health 24, 124–131. 11199279 PMC6713016

[B82] Stahel P. F. Smith W. R. Bruchis J. Rabb C. H. (2008). Peroxisome proliferator-activated receptors: “key” regulators of neuroinflammation after traumatic brain injury. PPAR Res. 2008:538141. 10.1155/2008/538141 18382619 PMC2276625

[B83] Tajuddin N. Moon K. H. Marshall S. A. Nixon K. Neafsey E. J. Kim H. Y.. (2014). Neuroinflammation and neurodegeneration in adult rat brain from binge ethanol exposure: abrogation by docosahexaenoic acid. PLoS ONE 9:e101223. 10.1371/journal.pone.0101223 25029343 PMC4100731

[B84] Udomuksorn W. Mukem S. Kumarnsit E. Vongvatcharanon S. Vongvatcharanon U. (2011). Effects of alcohol administration during adulthood on parvalbumin and glial fibrillary acidic protein immunoreactivity in the rat cerebral cortex. Acta Histochem. 113, 283–289. 10.1016/j.acthis.2009.11.001 20056265

[B85] Vetreno R. P. Crews F. T. (2012). Adolescent binge drinking increases expression of the danger signal receptor agonist HMGB1 and Toll-like receptors in the adult prefrontal cortex. Neuroscience 226, 475–488. 10.1016/j.neuroscience.2012.08.046 22986167 PMC3740555

[B86] Vinod K. Y. Yalamanchili R. Xie S. Cooper T. B. Hungund B. L. (2006). Effect of chronic ethanol exposure and its withdrawal on the endocannabinoid system. Neurochem. Int. 49, 619–625. 10.1016/j.neuint.2006.05.002 16822589

[B87] Vogel C. Marcotte E. M. (2012). Insights into the regulation of protein abundance from proteomic and transcriptomic analyses. Nat. Rev. Genet. 13, 227–232. 10.1038/nrg3185 22411467 PMC3654667

[B88] Wilson J. Watson W. P. Little H. J. (1998). CCK(B) antagonists protect against anxiety-related behaviour produced by ethanol withdrawal, measured using the elevated plus maze. Psychopharmacology 137, 120–131. 10.1007/s002130050601 9629998

[B89] Zeigler D. W. Wang C. C. Yoast R. A. Dickinson B. D. Mccaffree M. A. Robinowitz C. B.. (2005). The neurocognitive effects of alcohol on adolescents and college students. Prev. Med. 40, 23–32. 10.1016/j.ypmed.2004.04.044 15530577

