# Supplementary material for: Effects of Intermittent Alcohol Exposure on Emotion and Cognition: A Potential Role for the Endogenous Cannabinoid System and Neuroinflammation
Source: Front Behav Neurosci. 2017 Feb 7;11:15. doi: 10.3389/fnbeh.2017.00015 (PMC5293779; doi:10.3389/fnbeh.2017.00015)
Supplement: Supplementary file 1 [file Table1.DOCX]

**Table S1.** Adjustment for Multiple Comparisons (Benjamini–Hochberg false discovery rate approach) in Figure 5

| **Prefrontal Cortex (Figure 5A) Alcohol vs. Control** | | | | | | |
| --- | --- | --- | --- | --- | --- | --- |
| **Gene** | **Degrees of Freedom** | **t-statistic** | ***p*-value** | ***q*-value** | ***p<q*** | **Adjusted Significance** |
| *Cnr1* | 14 | 0.984 | 0.3419 | 0.0375 | False |  |
| *Cnr2* | 14 | 0.395 | 0.3948 | 0.0438 | False |  |
| *Ppara* | 7 | 4.959 **^w^** | **0.0016** | 0.0250 | True | Yes |
| *Napepld* | 14 | 6.124 | **<0.0001** | 0.0188 | True | Yes |
| *Dagla* | 7 | 12.470 **^w^** | **<0.0001** | 0.0063 | True | Yes |
| *Daglb* | 7 | 9.740 **^w^** | **<0.0001** | 0.0125 | True | Yes |
| *Faah* | 14 | 2.530 | **0.0240** | **0.0313** | True | Yes |
| *Mgll* | 14 | 0.3928 | 0.7004 | 0.0500 | False |  |
| **Striatum (Figure 5B) Alcohol vs. Control** | | | | | | |
| **Gene** | **Degrees of Freedom** | **t-statistic** | ***p*-value** | ***q*-value** | ***p<q*** | **Adjusted Significance** |
| *Cnr1* | 7 | 6.658 **^w^** | **0.0003** | **0.0125** | True | Yes |
| *Cnr2* | 7 | 2.940 **^w^** | **0.0217** | 0.0188 | False | No |
| *Ppara* | 8 | 14.55 **^w^** | **<0.0001** | 0.0063 | True | Yes |
| *Napepld* | 14 | 1.010 | 0.3296 | 0.0438 | False |  |
| *Dagla* | 8 | 0.137 **^w^** | 0.8941 | 0.0500 | False |  |
| *Daglb* | 14 | 1.414 | 0.1792 | 0.0250 | False |  |
| *Faah* | 14 | 1.093 | 0.2928 | 0.0313 | False |  |
| *Mgll* | 14 | 1.029 | 0.3209 | 0.0375 | False |  |
| **Amygdala (Figure 5C) Alcohol vs. Control** | | | | | | |
| **Gene** | **Degrees of Freedom** | **t-statistic** | ***p*-value** | ***q*-value** | ***p<q*** | **Adjusted Significance** |
| *Cnr1* | 14 | 4.122 | **0.0010** | 0.0063 | True | Yes |
| *Cnr2* | 14 | 0.319 | 0.7541 | 0.0500 | False |  |
| *Ppara* | 14 | 2.031 | 0.0617 | 0.0250 | False |  |
| *Napepld* | 9 | 2.692 **^w^** | **0.0247** | 0.0188 | False | No |
| *Dagla* | 14 | 3.441 | **0.0040** | **0.0125** | True | Yes |
| *Daglb* | 14 | 1.985 | 0.0671 | 0.0313 | False |  |
| *Faah* | 8 | 0.721 **^w^** | 0.4914 | 0.0438 | False |  |
| *Mgll* | 14 | 1.214 | 0.2449 | 0.0375 | False |  |
| **Hippocampus (Figure 5D) Alcohol vs. Control** | | | | | | |
| **Gene** | **Degrees of Freedom** | **t-statistic** | ***p*-value** | ***q*-value** | ***p<q*** | **Adjusted Significance** |
| *Cnr1* | 9 | 0.231 **^w^** | 0.8227 | 0.0500 | False |  |
| *Cnr2* | 7 | 3.960 **^w^** | **0.0055** | 0.0063 | True | Yes |
| *Ppara* | 7 | 1.666 **^w^** | 0.1397 | 0.0313 | False |  |
| *Napepld* | 14 | 1.243 | 0.2344 | 0.0375 | False |  |
| *Dagla* | 14 | 2.811 | **0.0139** | 0.0125 | False | Yes |
| *Daglb* | 7 | 1.826 **^w^** | 0.1106 | 0.0250 | False |  |
| *Faah* | 14 | 1.103 | 0.2887 | 0.0438 | False |  |
| *Mgll* | 9 | 3.020 **^w^** | **0.0145** | **0.0188** | True | Yes |
| **(^w^)** Welch´s t-test for unequal variances  *q*-value: False discovery rate (FDR) adjusted *p*-value  In red the FDR adjusted significance level | | | | | | |
