# Supplementary material for: Effects of Intermittent Alcohol Exposure on Emotion and Cognition: A Potential Role for the Endogenous Cannabinoid System and Neuroinflammation
Source: Front Behav Neurosci. 2017 Feb 7;11:15. doi: 10.3389/fnbeh.2017.00015 (PMC5293779; doi:10.3389/fnbeh.2017.00015)
Supplement: Supplementary file 2 [file Table2.DOCX]

**Table S2.** Adjustment for Multiple Comparisons (Benjamini–Hochberg false discovery rate approach) in Figure 6

| **Amygdala (Figure 6A) Alcohol vs. Control** | | | | | | |
| --- | --- | --- | --- | --- | --- | --- |
| **Gene** | **Degrees of Freedom** | **t-statistic** | ***p*-value** | ***q*-value** | ***p<q*** | **Adjusted Significance** |
| *Crh* | 14 | 0.3853 | 0.7058 | 0.0429 | False |  |
| *Crhr1* | 14 | 1.067 | 0.3042 | 0.0286 | False |  |
| *Crhr2* | 14 | 1.877 | 0.0816 | 0.0214 | False |  |
| *Npy* | 9 | 2.398 ^w^ | **0.0400** | 0.0143 | False | No |
| *Npy1r* | 14 | 0.7926 | 0.4412 | 0.0357 | False |  |
| *Npy2r* | 14 | 3.071 | **0.0066** | **0.0071** | True | Yes |
| *Npy5r* | 14 | 0.1162 | 0.9091 | 0.0500 | False |  |
| **Hippocampus (Figure 6B) Alcohol vs. Control** | | | | | | |
| **Gene** | **Degrees of Freedom** | **t-statistic** | ***p*-value** | ***q*-value** | ***p<q*** | **Adjusted Significance** |
| *Crh* | 14 | 3.756 | **0.0021** | **0.0143** | True | Yes |
| *Crhr1* | 14 | 0.8497 | 0.4098 | 0.0357 | False |  |
| *Crhr2* | 14 | 0.3763 | 0.7123 | 0.0500 | False |  |
| *Npy* | 14 | 2.224 | **0.0431** | 0.0286 | False | No |
| *Npy1r* | 14 | 0.6402 | 0.5324 | 0.0429 | False |  |
| *Npy2r* | 14 | 6.600 | **<0.0001** | 0.0071 | True | Yes |
| *Npy5r* | 14 | 2.463 | **0.0273** | 0.0214 | False | No |
| **(^w^)** Welch´s t-test for unequal variances  *q*-value: False discovery rate (FDR) adjusted *p*-value  In red the FDR adjusted significance level | | | | | | |
