# Supplementary material for: Effects of Intermittent Alcohol Exposure on Emotion and Cognition: A Potential Role for the Endogenous Cannabinoid System and Neuroinflammation
Source: Front Behav Neurosci. 2017 Feb 7;11:15. doi: 10.3389/fnbeh.2017.00015 (PMC5293779; doi:10.3389/fnbeh.2017.00015)
Supplement: Supplementary file 3 [file Table3.DOCX]

**Table S3.** Adjustment for Multiple Comparisons (Benjamini–Hochberg false discovery rate approach) in Figure 7

| **Prefrontal Cortex (Figure 7A) Alcohol vs. Control** | | | | | | | | | | |
| --- | --- | --- | --- | --- | --- | --- | --- | --- | --- | --- |
| **Gene** | **Degrees of Freedom** | | **t-statistic** | ***p*-value** | ***q*-value ^BH^** | | | ***p<q*** | | **Adjusted Significance** |
| *Tnf* | 9 | | 3.547 **^w^** | **0.0062** | 0.0333 | | | True | | Yes |
| *Tlr4* | 14 | | 12.50 | **<0.0001** | 0.0083 | | | True | | Yes |
| *Ptgs2* | 14 | | 2.670 | **0.0183** | **0.0417** | | | True | | Yes |
| *Rela* | 14 | | 4.174 | **0.0009** | 0.0250 | | | True | | Yes |
| *Gfap* | 7 | | 9.075 **^w^** | **<0.0001** | 0.0167 | | | True | | Yes |
| *Aif1* | 14 | | 1.469 | 0.1638 | 0.0500 | | | False | |  |
| **Striatum (Figure 7B) Alcohol vs. Control** | | | | | | | | | | |
| **Gene** | **Degrees of Freedom** | | **t-statistic** | ***p*-value** | ***q*-value ^BH^** | | | ***p<q*** | | **Adjusted Significance** |
| *Tnf* | 14 | | 1.458 | 0.1670 | 0.0417 | | | False | |  |
| *Tlr4* | 14 | | 0.822 | 0.4247 | 0.0500 | | | False | |  |
| *Ptgs2* | 14 | | 4.339 | **0.0007** | 0.0083 | | | True | | Yes |
| *Rela* | 9 | | 5.909 **^w^** | **0.0009** | **0.0167** | | | True | | Yes |
| *Gfap* | 14 | | 2.477 | **0.0266** | 0.0250 | | | False | | No |
| *Aif1* | 7 | | 2.506 **^w^** | **0.0406** | 0.0333 | | | False | | No |
| **Amygdala (Figure 7C) Alcohol vs. Control** | | | | | | | | | | |
| **Gene** | **Degrees of Freedom** | | **t-statistic** | ***p*-value** | ***q*-value ^BH^** | | ***p<q*** | | | **Adjusted Significance** |
| *Tnf* | 14 | | 0.687 | 0.5032 | 0.0333 | | False | | |  |
| *Tlr4* | 14 | | 0.386 | 0.7050 | 0.0417 | | False | | |  |
| *Ptgs2* | 14 | | 0.166 | 0.8705 | 0.0500 | | False | | |  |
| *Rela* | 7 | | 2.087 **^w^** | 0.0753 | 0.0250 | | False | | |  |
| *Gfap* | 14 | | 1.997 | 0.0657 | 0.0167 | | False | | |  |
| *Aif1* | 8 | | 3.451 **^w^** | **0.0079** | **0.0083** | | True | | | Yes |
| **Hippocampus (Figure 7D) Alcohol vs. Control** | | | | | | | | | | |
| **Gene** | | **Degrees of Freedom** | **t-statistic** | ***p*-value** | | ***q*-value ^BH^** | | | ***p<q*** | **Adjusted Significance** |
| *Tnf* | | 7 | 1.311 **^w^** | 0.2311 | | 0.0417 | | | False |  |
| *Tlr4* | | 14 | 0.598 | 0.5594 | | 0.0500 | | | False |  |
| *Ptgs2* | | 14 | 2.724 | **0.0165** | | 0.0167 | | | True | Yes |
| *Rela* | | 14 | 1.952 | 0.0713 | | 0.0333 | | | False |  |
| *Gfap* | | 14 | 2.741 | **0.0159** | | 0.0083 | | | True | Yes |
| *Aif1* | | 8 | 2.844 **^w^** | **0.0217** | | **0.0250** | | | True | Yes |
| **(^w^)** Welch´s t-test for unequal variances  *q*-value: False discovery rate (FDR) adjusted *p*-value  In red the FDR adjusted significance level | | | | | | | | | | |
